# Targeting histone deacetylases in head and neck squamous cell carcinoma: molecular mechanisms and therapeutic targets

**DOI:** 10.1186/s12967-024-05169-9

**Published:** 2024-05-03

**Authors:** Mengchen Xu, Yiming Hou, Na Li, Wenqian Yu, Lei Chen

**Affiliations:** 1 Shandong Key Laboratory of Oral Tissue Regeneration & Shandong Engineering Laboratory for Dental Materials and Oral Tissue Regeneration, Department of Orthodontics, School and Hospital of Stomatology, Shandong Provincial Clinical Research Center for Oral Diseases, Cheeloo College of Medicine, Shandong University, Jinan, 250012 Shandong China; 2 Department of Otolaryngology-Head and Neck Surgery, Shandong Provincial ENT Hospital, Cheeloo College of Medicine, Shandong University, Jinan, 250022 Shandong China; 3 Center of Clinical Laboratory, Shandong Second Provincial General Hospital, Jinan, 250022 Shandong China; 4 Research Center of Translational Medicine, Department of Cardiac Surgery, Central Hospital Affiliated to Shandong First Medical University, Jinan, 250013 Shandong People’s Republic of China

**Keywords:** Head and neck squamous cell carcinoma, Histone deacetylases, Mechanism, Epigenetic therapy

## Abstract

The onerous health and economic burden associated with head and neck squamous cell carcinoma (HNSCC) is a global predicament. Despite the advent of novel surgical techniques and therapeutic protocols, there is an incessant need for efficacious diagnostic and therapeutic targets to monitor the invasion, metastasis and recurrence of HNSCC due to its substantial morbidity and mortality. The differential expression patterns of histone deacetylases (HDACs), a group of enzymes responsible for modifying histones and regulating gene expression, have been demonstrated in neoplastic tissues. However, there is limited knowledge regarding the role of HDACs in HNSCC. Consequently, this review aims to summarize the existing research findings and explore the potential association between HDACs and HNSCC, offering fresh perspectives on therapeutic approaches targeting HDACs that could potentially enhance the efficacy of HNSCC treatment. Additionally, the Cancer Genome Atlas (TCGA) dataset, CPTAC, HPA, OmicShare, GeneMANIA and STRING databases are utilized to provide supplementary evidence on the differential expression of HDACs, their prognostic significance and predicting functions in HNSCC patients.

**Graphical Abstract:**

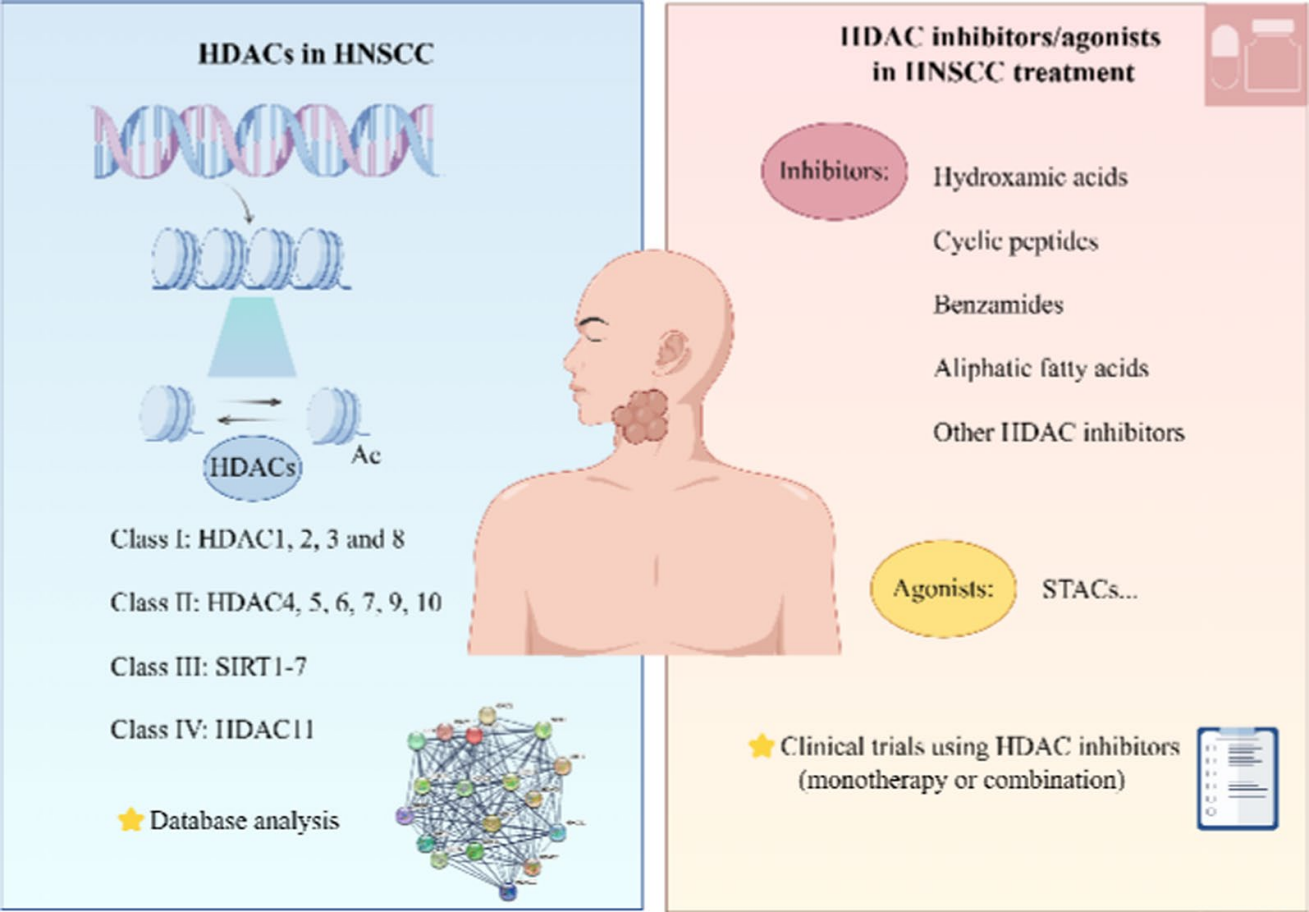

**Supplementary Information:**

The online version contains supplementary material available at 10.1186/s12967-024-05169-9.

## Introduction

**Fig. 1:**
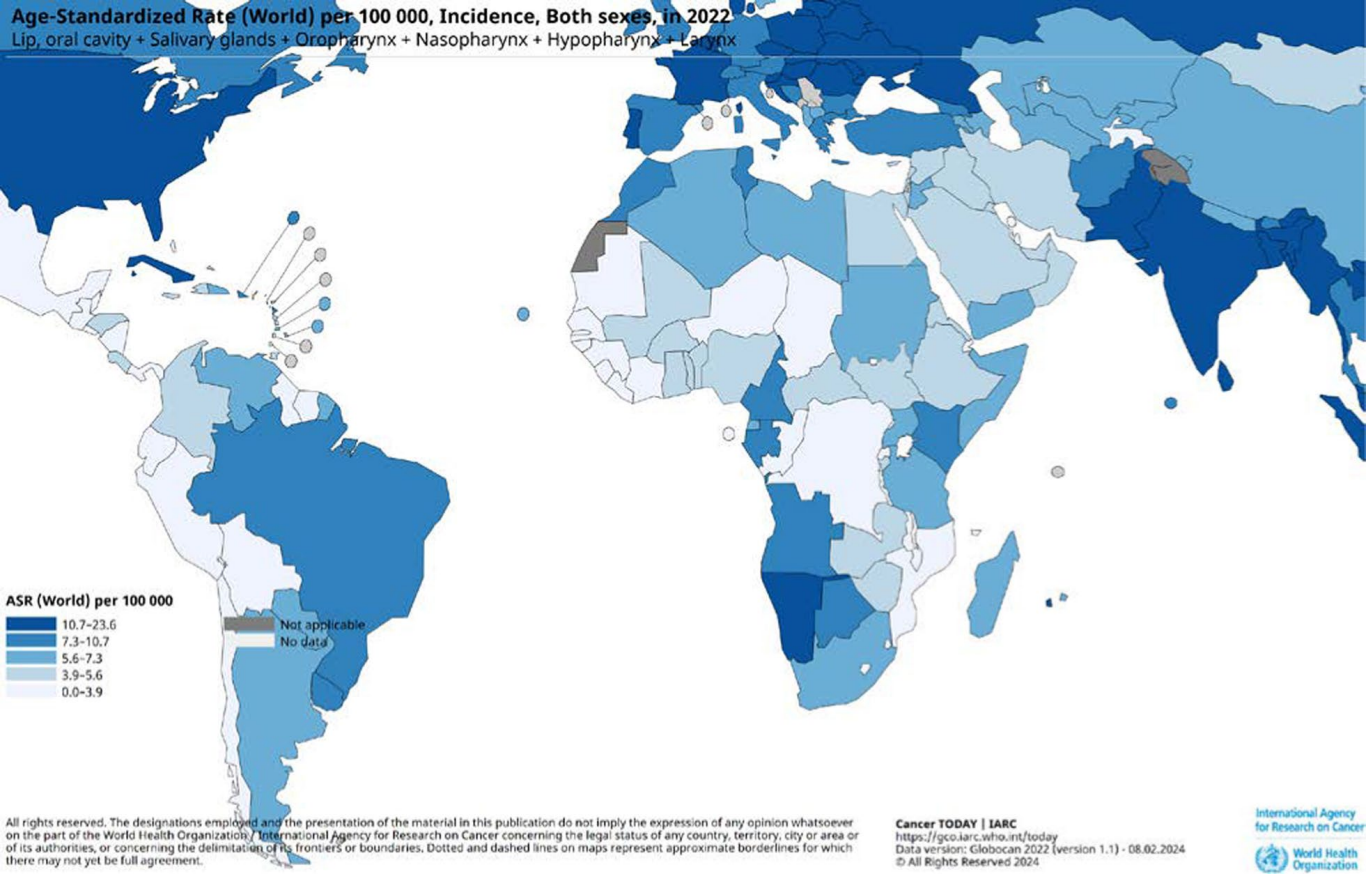
The global prevalence and distribution of HNSCC. The global prevalence of HNSCC is depicted through the estimated age-standardized rate for both genders. The data utilized is sourced from GLOBOCAN, 2022. The map was generated employing online mapping tool (https://gco.iarc.fr/today/online-analysis-map). The selected cancer sites encompass hypopharynx, larynx, lip, oral cavity, nasopharynx, salivary glands and oropharynx

Head and neck squamous cell carcinoma (HNSCC) originates from the epithelial lining of mucosal tissues of the head and neck [1]. It is a significant health concern worldwide, ranking sixth in cancer diagnosis frequency globally [2]. Notably high incidences of HNSCC are found in Russia, Southeast Asia, Australia and Western Europe (Fig. 1). HNSCC represents a multi-factorial, multi-stage immunosuppressive tumor group with molecular heterogeneity and complex tumor-host interactions [3]. The etiology of HNSCC remains elusive, contributing to increased morbidity and mortality rates and imposing substantial financial and health burdens [1]. Therapeutic interventions for HNSCC encompass surgery, radiotherapy (RT), chemotherapy and immunotherapy, with the choice guided by the TNM classification system as delineated by the Union for International Cancer Control/American Joint Committee on Cancer [4]. Innovative treatment modalities, such as laser surgery, photodynamic therapy and sonodynamic therapy, have made some progress in reducing morbidity and improving patient quality of life [5–7]. Nonetheless, the therapeutic efficacy of HNSCC management is still restricted. For patients with locally advanced HNSCC, chemoradiation therapy (CRT) is the mainstay treatment, however, approximately 50% experience disease recurrence within 5 years post-CRT [8]. Patients with recurrent or metastatic (R/M) HNSCC are mostly unable to receive curative treatment and palliative care is the mainstay of treatment, with limited options for targeted therapy and poor prognosis [9]. Immunotherapy with checkpoint inhibitors shows promise for better survival in some HNSCC patients [10], but only a mere fraction (less than 20%) [11], and the objective response rate for immunotherapy still requires enhancement [12]. Consequently, earnest endeavors remain indispensable to understand the pathogenesis of HNSCC and identify biomarkers for effective and safe treatments.

**Fig. 2:**
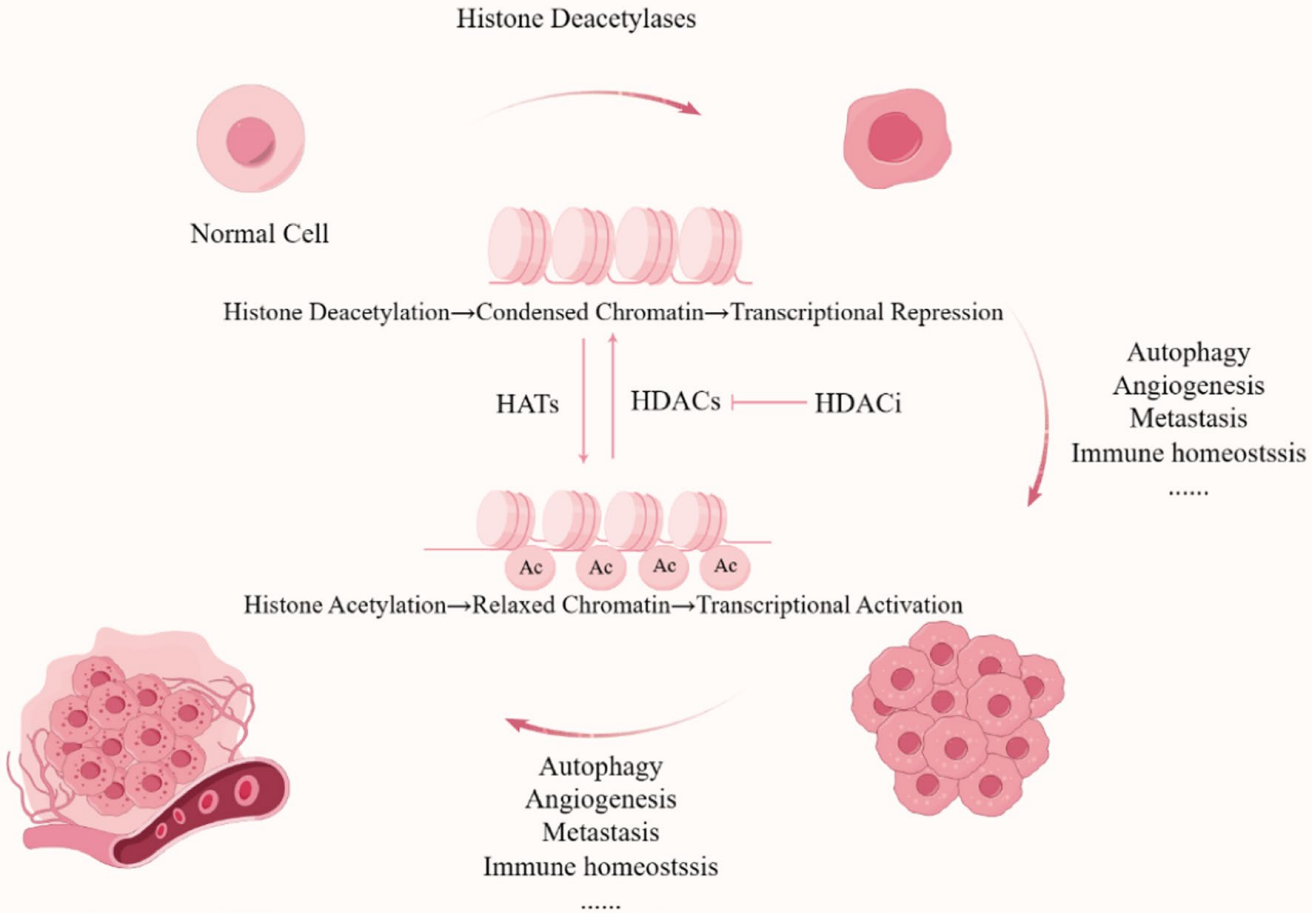
HDACs mediated-histone modifications affect key biological processes in HNSCC cell proliferation, angiogenesis, apoptosis and metastasis. Created with Figdraw (www.figdraw.com)

Recent research has provided empirical evidence supporting the significant involvement of epigenetic mechanisms in HNSCC [13, 14]. Epigenetics involves changes in heritable and reversible genetic characteristics without modifying the DNA sequence [15], including DNA methylation, histone modification, RNA editing, gene silencing and so forth [16]. Among these, histone acetylation assumes a pivotal position in protein expression and is linked to various pathologies such as tumorigenesis [17], aging [18], and bacterial infection [19], exhibiting important research significance. It is governed by enzymes called histone acetyltransferases (HATs) and histone deacetylases (HDACs) [20]. Acetylation of lysine residues on histones neutralizes their positive charge, relaxing chromatin structure and promoting gene transcription [20]. Deacetylation leads to gene silencing by reducing the accessibility of DNA to transcription factors (Fig. 2) [21]. Histone acetylation also performs a fundamental position in higher-order chromatin structure [22], nucleosome assembly [23] and preventing the spread of heterochromatin [24].

Deregulation of histone acetylation is linked to various illnesses and developmental processes [25, 26]. Excessive HDAC levels inhibit transcription by reducing the expression of p53, thermonuclear protein HSP90 and Smad family proteins, closely associated with the onset of carcinoma [27]. Moreover, elevated levels of classical HDACs have been linked to advanced disease progression and unfavorable patient outcomes [28]. When histone tails are highly acetylated, they adopt a more relaxed conformation with DNA, potentially leading to the overexpression of cancer-related genes [29]. Recent advancements show that HDACs can inhibit DNA repair, induce cell apoptosis and acetylate non-histone proteins [30, 31]. Although HDAC inhibitors and agonists impact gene activity and various cellular processes in tumor cells [30, 32], the specific roles of HDAC family members and their inhibitors or agonists in specific tumor models have not been thoroughly evaluated. Therefore, this article aims to discuss the roles and mechanisms of HDAC family members and their inhibitors and agonists in HNSCC, as well as their potential implications for clinical diagnosis and treatment.

## HDACs in HNSCC

Eighteen HDACs have been identified in humans and categorized into four types based on evolutionary evaluation and sequence homology analysis. Class I HDACs (HDAC1, 2, 3 and 8) share a similar sequence with the yeast protein Rpd3 and are usually localized in the nucleus, except for HDAC3 which can also be found in the cytoplasm. Class II HDACs, including HDAC4-7, 9 and 10, demonstrate significant sequence similarity with the Hda1 protein in yeast. They can translocate between the nucleus and cytoplasm in response to cellular signals and display distinct expression patterns in different cells or tissues. Class III represents a sirtuin protein family that shares a similar conservation with the silent statistics regulator 2 (Sir2) protein in yeast, encompassing SIRT1-7. Class IV is comprised solely of HDAC11, which exhibits moderate sequence homology between Rpd3 and Hda1 and is typically localized in the nucleus. Class I, II and IV HDACs are considered “classical” as they have highly homologous catalytic core domains that rely on zinc ions for catalysis. There is a wide range of biological functions due to significant variation in the sequences and structures of HDACs, particularly in their catalytic domains. In contrast, Class III HDACs belong to the NAD+ dependent Sir2 super-families and require zinc ions for deacetylase activity, but do not directly participate in the deacetylase reaction [33]. We further elucidate the roles of the pertinent HDAC classes in HNSCC, with Table 1 presenting a comprehensive summary of the corresponding data.

**Table 1:** HDACs associated with HNSCC

Classification	HDAC	Targets and mechanisms in HNSCC
Class I	HDAC1	Expression levels: up-regulated expression in LSCC samples [36] and HNSCC samples according to TCGA, CPTAC and HPA databases
		Cell proliferation: regulating the expression of PCNA and affecting cell proliferation by reducing Mrna stability [37]
		Invasion and metastases: interacting with RAR-α to regulate OSCC EMT and invasion through RAR-β [38]; associated with lymph node metastases and advanced clinical stage in LSCC [36]
		Cell death: acting as a central gene in autophagy process in HNSCC [39]
		Therapy resistance: down-regulated expression in cisplatin-resistant HNSCC, inversely associated with adhesion loss and drug resistance [40]
	HDAC2	Expression levels: positive HDAC2 nuclear staining was detected in 80/93 OSCC samples and 11/20 OED samples [41]
		Cell proliferation: associated with transcriptional repression and tumor maintenance in SCC [47]
		Invasion and metastases: maintaining HIF-1α stability, promoting cell invasion and metastases of oral cancer [44]; correlated with adhesion loss and aggressiveness, promoting the occurrence and depth of invasion in OSCC [32, 42]
		Cell death: playing a significant role in trichodermin [45] orγ-bisabolene [46] induced apoptosis in HNSCC
	HDAC3	Immunosuppression: acting as the target of PKM2-mediated immunosuppression in HNSCC [49]
	HDAC8	Expression levels: up-regulated expression in OSCC [44] and HNSCC samples according to TCGA database
		Cell death: knocking down HDAC8 induce apoptotic cell death through caspase activation and autophagy in OSCC [51, 52]
Class II	HDAC4	Expression levels: up-regulated expression in NPC [55], Cal27 [56] and ESCC [57] cells, but the TCGA, CPTAC and HPA databases revealed a down-regulated expression in HNSCC, indicating that HDAC4 may possess dual roles in cancer development
		Cell proliferation: promoting proliferation and metastasis of NPC cells by upregulating TYK2-STAT1 phosphorylation through HDAC4/LHPP axis [55]; promoting cell proliferation through facilitating G1/S cell cycle in EC cells, while upregulating CDK2/4 and CDK-dependent Rb phosphorylation [57]
		Cell death: HDAC4 deletion leads to the apoptosis of HNSCC cells [51]
		Prognostic significance: poor overall survival and progression-free survival are associated with high HDAC4 expression [51]
		Therapy resistance: HDAC4 silencing promotes microRNA-146a, contributing to radio-sensitization of esophageal cancer [59]; trail-resistant HNSCC cells are sensitized by loss of HDAC4 [44]; influencing the cytotoxicity of HDAC inhibitors [61]
	HDAC5	Expression levels: TCGA, CPTAC and HPA databases showed the Mrna expression of HDAC5 was down-regulated in HNSCC samples
		Cell death: playing roles in regulating EBV lytic replication and cell death [56]
	HDAC7	Cell proliferation: HDAC7 knockdown resulted in growth suppression through G2/M arrest in MEC [63]; targeting of HDAC7 could potentially disrupt the miR-4465-EphA2 pathway and impede the progression of NPC [64]
		Invasion and metastases: target of miR-140-5p mediated invasion and migration suppression of TSCC [62]
		Cell death: HDAC7 knockdown in MEC promote the apoptosis and autophagy process [63]
	HDAC9	Expression levels: up-regulated expression in OSCC [34]
		Cell proliferation: promoting OSCC carcinogenesis by targeting transcription factor MEF2D and pro-apoptotic factor NR4A1/Nur77 [34]
		Cell death: low expression of HDAC9 lead to G0/G1 cycle arrest in OSCC cells and increase cell apoptosis [34]
	HDAC6	Expression levels: down-regulated expression according to TCGA, CPTAC and HPA databases, however, Sakuma et al. [69] observed HDAC6 expression increased in OSCC
		Cell proliferation: miR-433 downregulate HDAC6 expression by targeting its 3′UTR, leading to the inhibition of OSCC cell growth [71]; miR-206 impede proliferation of HNSCC cells through targeting HDAC6 through PTEN/AKT/Mtor signal [72]
		Cell death: related to autophagy and mediates the regulation between ER stress and autophagy [34]
		Prognostic significance: negative associations between HDAC6 expression and the OA of OSCC patients [73]; significant differences in HDAC6 expression between early stage and advanced stage OSCC samples [69]
		Therapy resistance: pharmacological target for overcoming chemotherapy resistance and preventing OSCC recurrence [34]
	HDAC10	Expression levels: TCGA database analysis showed HDAC10 expression was increased in HNSCC samples
Class III	SIRT1	Expression levels: functioning as a dual-role factor in HNSCC; SIRT1 expression significantly decreased in HNSCC according to TCGA database, however, SIRT1 expression was significantly lower in SCC-9 and SCC-25 [81]
		Cell proliferation: binding to the promoter of TGF-β, impeding CBP/p300-mediated acetylation, leading to transcriptional suppression, exerting inhibitory effects on the proliferation of OSCC [78]
		Invasion and metastases: inhibiting vimentin and N-cadherin expression, suppressing Smad2/3 phosphorylation and Smad4 deacetylation, impeding the EMT process in OSCC [82–84]
		Cell death: capsaicin hinders SIRT1 activity, augmenting acetylation of unc-51-like autophagy-activating kinase 1 and instigating autophagy in OSCC [90]
		Prognostic significance: potential as a valuable prognostic assessment tool according to Kaplan–Meier analysis and clinical observation [86]
		Therapy resistance: enhance cisplatin resistance of OSCC [88]; downregulation of SIRT1 resulted in PI3K/AKT/Mtor pathway inhibition, reversing the radio-resistance of ECA-109 cells [88]; SIRT1 inducing EBV-miR-BART4 mediated stemness and cisplatin resistance in NPC carcinoma side cells [89]
	SIRT2	Expression levels: TCGA, CPTAC and HPA databases showed decreased SIRT2 Mrna expression in HNSCC
		Prognostic significance: Kaplan–Meier survival analysis highlight the potential value of SIRT2 in validating prognosis
		Lymph angiogenesis: playing an important role in HNSCC lymph angiogenesis [93]
	SIRT3	Expression levels: up-regulated expression in OSCC [94], however, enzyme deacetylation is reduced, indicating presence of variants such as p.Val208Ile [95]
		Cell proliferation: SIRT3 downregulation significantly restrains proliferation in ESCC [96] and OSCC [94]
		Invasion and metastases: mitigating augmented oxidative stress induced by miR-31, thereby promoting migration and invasion in OSCC [99, 100]
		Cell death: participating in redox homeostasis, energy metabolism and mitochondrial fission, inducing intrinsic apoptosis [94]
		Therapy resistance: SIRT3 downregulation heightens OSCC cell sensitivity to radiation and cisplatin treatments [101]
	SIRT4	Expression levels: down-regulated expression in HNSCC according to TCGA
		Invasion and metastases: SIRT4 downregulation associated with a more aggressive tumor phenotype and unfavorable prognosis [104]
	SIRT5	Cell proliferation and metastasis: inhibiting Warburg effect, countering ROS damage and inhibiting cell proliferation and metastasis [88]
		Therapy resistance: SIRT5 exhibit carcinogenic characteristics, leading to chemotherapy and radiotherapy resistance [88]
	SIRT6	Expression levels: genetic expression profiling of 34 HNSCC patients [107], OSCC tissues [108] and TCGA database both revealed a significant upregulation of SIRT6 in cancer group; however, 82 cases of OLP and 77 cases of OSCC were examined, revealing significantly lower expression of SIRT6 in OSCC lesions [108]
		Pro-differentiation: down-regulation of SIRT6 replicates the pro-differentiation effects of miR-34a in SCCs [109]
		Cell death: increasing ROS expression, promote apoptosis, modulate telomere maintenance in OSCC [88]
		Prognostic significance: associated with shorter overall survival [28]
	SIRT7	Invasion and metastases: inhibiting EMT, invasion, migration and metastasis in OSCC cells [112]; miR-770 facilitating migration and invasion of OSCC cells through the activation of SIRT7/Smad4 pathway [113]; SIRT7 expression positive associated with stromal lymphocytic infiltration and invasion depth in OSCC [108]
Class IV	HDAC11	Expression levels: TCGA database showed a decreasing trend of HDAC11 expression in HNSCC samples

**Fig. 3:**
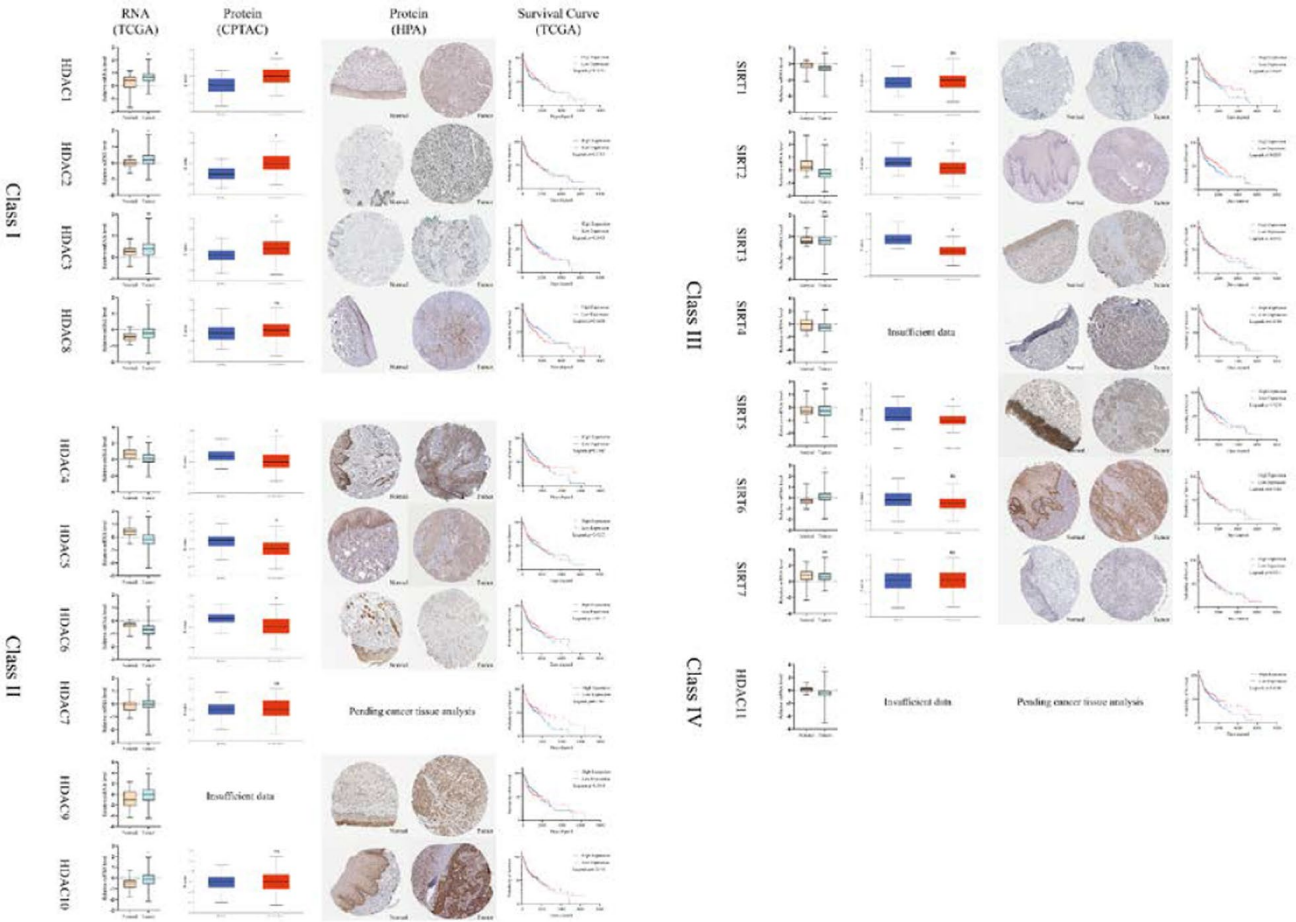
Relative mRNA and protein expression comparisons of different HDAC family in tumor and normal tissues. The two-tailed unpaired t-test was employed to evaluate the relative mRNA expression levels of different HDAC family members using TCGA database, which includes RNA sequencing data from 520 HNSCC samples and 44 normal samples. The protein levels of different HDACs were analyzed through CPTAC database, encompassing 108 HNSCC samples and 71 normal samples, with validation by HPA database. The Kaplan–Meier plotter highlighted the prognostic importance of HDAC mRNA expressions in HNSCC patients. Data represent the mean ± SD. *p < 0.05

**Fig. 4:**
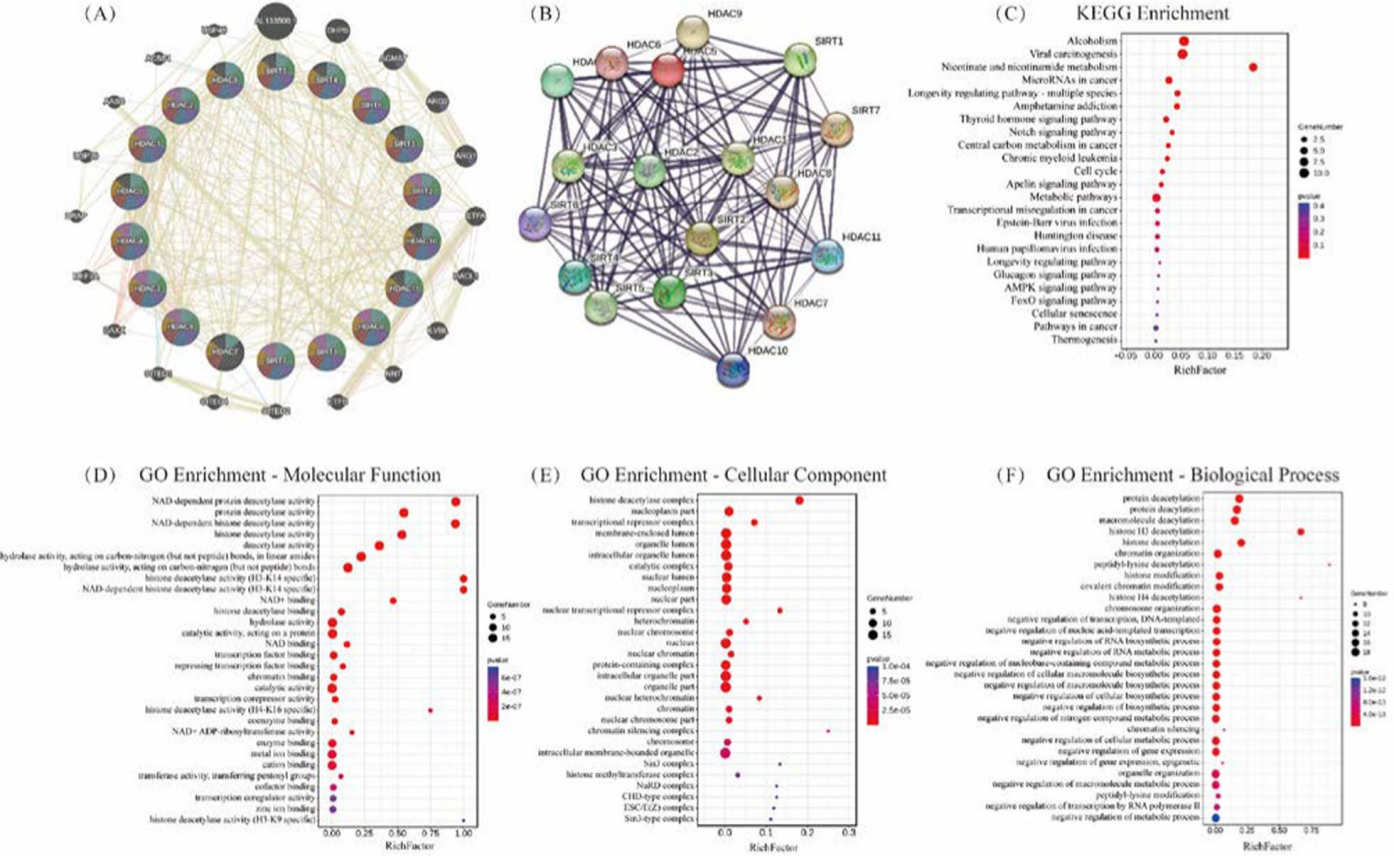
The predictive functions and pathways of HDACs along with their neighboring genes in HNSCC. **A** Network of HDACs and their neighboring genes was constructed using GeneMANIA (http://genemania.org). **B** HDAC family protein–protein interaction (PPI) network by STRING (https://string-db.org). Kyoto Encyclopedia of Genes and Genomes (KEGG) pathway analyses of HDAC genes was shown in **C** (https://www.omicshare.com). Gene ontology (GO) enrichment analysis of HDAC genes was conducted based on molecular function (**D**), cellular component (**E**) and biological process (F) (https://www.omicshare.com)

The Cancer Genome Atlas (TCGA) database (https://www.cancer.gov/ccg/research/genome-sequencing/tcga) was employed to examine HDAC expression in HNSCC, to investigate the association between HDACs and clinical parameters and to predict how HDACs may contribute to tumor growth. 520 samples of HNSCC RNA Seq statistics and clinical data, along with 44 normal tissue samples, have been procured from the TCGA database to investigate HDAC expression and its impact on patient overall survival (OA) (Fig. 3). Protein analysis has the potential to complement RNA data. The protein levels of different HDACs were analyzed by CPTAC (https://ualcan.path.uab.edu/analysis-prot.html) (108 HNSCC samples and 71 normal samples) and validated by HPA (https://www.proteinatlas.org/). The study investigated the predictive functions and pathways of HDACs and their neighboring genes in HNSCC (Fig. 4). The network of HDACs and their neighboring genes was constructed using GeneMANIA (http://genemania.org) (Fig. 4A), while protein–protein analyses were conducted with STRING (https://string-db.org) to identify their functions (Fig. 4B). The Kyoto Encyclopedia of Genes and Genomes (KEGG) pathway analyses of HDAC genes were obtained from OmicShare (https://www.omicshare.com) (Fig. 4C). Furthermore, gene ontology (GO) enrichment analysis of HDAC genes was performed, considering molecular function (Fig. 4D), cellular component (Fig. 4E) and biological process (Fig. 4F), also sourced from OmicShare.

### Class I HDACs

HDAC1-3 exhibit robust deacetylase activity by interacting with other proteins to form co-inhibitory complexes [34]. HDAC1 and HDAC2 form the catalytic core of multiple inhibitory complexes, such as Sin3A complex, nucleosome remodeling deacetylase (NuRD) complex and corepressor of RE1-silencing transcription factor (CoREST) complex. HDAC3 plays a crucial role in the assembly of the silencing mediator of retinoic acid and thyroid hormone receptors/nuclear receptor corepressor (SMRT/NcoR) complex, activating deacetylase activity of HDAC1-3 and facilitating transcriptional silencing at specific loci through interactions with regulatory proteins. HDAC8, on the other hand, exhibits substantial histone deacetylase activity, suggesting a potential capacity for independent functioning [35].

The TCGA, CPTAC and HPA databases demonstrated a significant elevation in HDAC1 expression in HNSCC (*p* < *0.05*) (Fig. 3). Zhao et al. [36] examined HDAC1 expression in 90 cases of laryngeal squamous cell carcinoma (LSCC), 30 cases of adjacent non-tumor tissue from laryngeal polyps and 30 cases of laryngeal polyp tissue. Researchers observed a frequent overexpression of HDAC1 in LSCC samples, particularly in young males with poorly differentiated tumors and lymph node metastases. Inhibiting HDAC1 with PCI-24781 or siRNA reduced oral squamous cell carcinoma (OSCC) cell proliferation by affecting proliferating cell nuclear antigen (PCNA) regulation and mRNA stability. HDAC1 also acts as a negative regulator of miR-154-5p, attenuating the reduction of PCNA and the cell proliferation inhibition induced by PCI-24781 [37]. HDAC1 participates in HNSCC adhesion loss and invasion. CCL28 signaling increases retinoic acid receptor (RAR)-β expression by diminishing RAR-α and HDAC1 interaction, negatively regulating epithelial-mesenchymal transition (EMT) and bone invasion of OSCC [38]. Kondapuram et al. [39] proposed that HDAC1 acted as a central gene in autophagy in various cancer types, including HNSCC, which exhibited a strong correlation with patient survival, becoming a promising therapeutic target. However, several studies have demonstrated a negative correlation between HDAC1 and drug resistance in HNSCC. Its expression declined in both intrinsic and acquired cisplatin-resistant HNSCC cells, indicating an intricate role of HDAC1 in the cancer microenvironment [40].

Chang et al. [41] found positive HDAC2 nuclear staining was detected in 80/93 OSCC samples and 11/20 oral epithelial dysplasia (OED) samples and the labeling index for HDAC2 staining increased substantially from OED (25.8 ± 26.5%) to OSCCs (59.8 ± 28.5%) (*p* < *0.001*) [41]. HDAC2 protein expression was also elevated in advanced, giant tumor size or lymph node metastasis-positive tumors, associated with adhesion loss and invasiveness [42, 43]. HDAC2 could maintain HIF-1α stability, promoting oral cancer progression through enhanced cell invasion and migration [44]. HDAC2 plays a significant role in the apoptosis of HNSCC. Trichodermin suppressed the proliferation of OSCC cells by inducing apoptosis through mitochondrial dysfunction and activating HDAC2-related pathway [45]. Additionally, γ-bisabolene could trigger p53-mediated apoptosis in human OSCC by inhibiting HDAC2 and activating ERK1/2 [46]. HDAC1 and 2 can also form a complex with p63 to repress transcription and maintain tumors in SCC [47].

HDAC3, an essential enzyme involved in the maintenance of chromatin structure and genomic stability, plays a crucial role in various cellular processes such as DNA replication, DNA damage repair and chromatin remodeling [48]. Increased levels of pyruvate kinase isozyme type M2 (PKM2) facilitated the production of lactate, consequently hindering the formation of the HDAC3 inhibitory transcription complex and facilitating the transcription of Galectin-9, leading to immunosuppression. It was worth mentioning that RFPG966, a specific inhibitor of HDAC3, significantly enhanced colony formation and augmented cell invasion and migration, indicating its potential as a therapeutic intervention [49].

Inhibiting HDAC8 has emerged as a promising cancer therapeutic strategy [50]. The TCGA database analysis revealed high HDAC8 expression in HNSCC tissues, consistent with the findings of Ahn et al. [51] in OSCC. Apicidin effectively suppressed murine OSCC cell growth in vitro and in vivo by inhibiting HDAC8 expression, leading to a significant 46% reduction in tumor growth within 14 days [52]. Moreover, knocking down HDAC8 has been shown to induce apoptotic cell death through caspase activation and autophagy [51, 52].

#### Class II HDACs

Class II HDACs can be further categorized into two subclasses: Class IIa and Class IIb. Class IIa HDACs, including HDAC4, HDAC5, HDAC7 and HDAC9, exhibit tissue-specific abundance in skeletal muscle, brain and heart [53]. These HDACs primarily modulate gene expression by shuttling between the nucleus and cytoplasm. Class IIb HDACs, specifically HDAC6 and HDAC10, predominantly localize in the cytoplasm and exert deacetylation effects on non-histone proteins. Class IIa HDACs have weaker deacetylase activity due to a tyrosine mutation in the catalytic site [53]. Interestingly, the deacetylase activity of HDAC3 can be activated by HDAC4, HDAC5 and HDAC7 through the interaction of SMRT/NCoR complex in vivo [54], suggesting that class IIa HDACs may function as regulatory factors.

HDAC4 was overexpressed in nasopharyngeal carcinoma (NPC) cells and promoted their proliferation and metastasis by upregulating TYK2-STAT1 phosphorylation through HDAC4/LHPP signal axis [55]. HDAC4 overexpression in Cal27 cells also led to increased proliferation compared to control cells [56]. Additionally, HDAC4 was overexpressed in esophageal squamous cell carcinoma (ESCC), where its overexpression was closely linked to increased tumor grade, advanced clinical stage and unfavorable survival outcomes [57]. Mechanistically, HDAC4 promoted cell proliferation and facilitated G1/S cell cycle progression in EC cells by suppressing p21 and p27, while simultaneously upregulating CDK2/4 and CDK-dependent Rb phosphorylation [57]. However, analysis of the TCGA, CPTAC and HPA databases revealed a down-regulation of mRNA expression for HDAC4 in HNSCC samples, suggesting it may have dual roles in cancer development. Cheng et al. [58] reported a correlation between high HDAC4 levels and diminished overall survival as well as progression-free survival rates in HNSCC. HDAC4 silencing promoted microRNA-146a, enhancing radio-sensitization of esophageal cancer [59]. Loss of HDAC4 sensitized TRAIL-resistant HNSCC cells to apoptosis [60]. The expression status of HDAC4 also has a certain effect on the cytotoxicity of HDAC inhibitors [61].

The TCGA database showed decreased mRNA expression of HDAC5 in HNSCC samples. The protein analysis using CPTAC and HPA databases has the potential to complement RNA data. Andrographolide could inhibit Epstein Barr virus (EBV) reactivation, linked to viral transmission and oncogenesis in HNSCC cells [56]. Andrographolide also boosted HDAC5 and HDAC9 expression in EBV-positive cells, interacting with MEF2D, specific protein (Sp) 1 and Sp3 to regulate EBV lytic replication and cell death [56]. While HDAC5 played a relatively minor role in the cytotoxicity of HDAC inhibitors. In HDAC5 overexpression clones, Bortezomib demonstrated an IC_50_ of 5 nM (nM), compared to 9 nM in control cells [61]. The IC_50_ for CHDI0039 ranged from 9.69 µM in control cells to 14.1 µM in cells with HDAC5 overexpression [61].

Confirmed as one of the specific endogenous targets of miR-140-5p, HDAC7 played a significant role in the miR-140-5p mediated invasion and migration inhibition in tongue squamous cell carcinoma (TSCC) cells [62]. HDAC7 knockdown in salivary mucoepidermoid carcinoma (MEC) cells led to growth suppression by G2/M arrest and the promotion of apoptosis and autophagy [63]. HDAC7 expression is significantly increased in NPC compared to normal tissues, positively correlated with NPC advancement and inversely associated with patient prognosis [64]. Targeting HDAC7 could potentially disrupt the miR-4465-EphA2 pathway and impede the progression of NPC [64]. However, there was no significant disparity in HDAC7 expression between normal and HNSCC samples based on the TCGA database. This finding can be attributed to the close association between HDAC7 function, its subcellular localization, and the prevailing environmental conditions [65]. Consequently, the role of HDAC7 may differ greatly across different cell types.

Another member of the class IIa HDACs, HDAC9, interacted with transcriptional suppressors and oncogenic proteins, modulating anti-tumor immunity by controlling CD8+ dendritic cells and T cell infiltration [66]. High expression of HDAC9 was seen in clinical OSCC samples and knocking it down induced G0/G1 cell cycle arrest and apoptosis in OSCC cells [67]. However, upregulation of HDAC9 facilitated OSCC development by interacting with transcription factor myocyte enhancer factor 2D (MEF2D) and pro-apoptotic factor NR4A1/Nur77 [67]. Rastogi et al. [68] identified HDAC9 as a target of miR-377 and demonstrated that miR-377 regulates HDAC9 as well as its pro-apoptotic target NR4A1/Nur77, thereby inhibiting cell growth, inducing apoptosis and reducing migration. The upregulation of HDAC2, HDAC9, SIRT1 and the HDAC1 nuclear distribution contributed to intrinsic resistance, promoting aggressiveness and loss of adhesion of HNSCC [43].

Furthermore, research has been done on the role of HDAC6, a class IIb HDACs, in HNSCC. The analysis of 520 samples unveiled a decrease in HDAC6 expression in HNSCC samples, but some studies have conflicting views. Sakuma et al. [69] observed the HDAC6 expression was elevated in OSCC compared to normal human oral keratinocytes (HOKs). The expression of HDAC6 has been linked to the proliferation and aggressiveness of HNSCC [70]. MiR-433 could downregulate HDAC6 expression by directly targeting its 3′UTR, leading to the inhibition of OSCC growth and metastasis [71]. Additionally, miR-206 inhibited HNSCC cell growth by targeting HDAC6 through the PTEN/AKT/mTOR signal [72]. Tseng et al. [73] found a negative correlation between HDAC6 levels and the overall survival of OSCC patients, indicating its potential as a prognostic biomarker. Researchers discovered that HDAC6 triggered IL-13 expression through AP-1, promoting M2 macrophage polarization in OSCC. This finding offered a novel approach for immune-related therapy in OSCC [73]. Furthermore, there was a notable difference in HDAC6 expression between early and advanced stage OSCC samples [69], suggesting a potential association between HDAC6 levels and tumor aggressiveness. Furthermore, HDAC6 has been implicated in autophagy and served as a critical regulator of cytoprotective response, bridging the connection between autophagy and Mubiquitin-proteasome system, mediating regulation between endoplasmic reticulum (ER) stress, autophagy and conferring chemoresistance in HNSCC [74]. Elevated levels of HDAC6 contributed to chemotherapy resistance in OSCC, making it a potential target for overcoming chemoresistance and preventing recurrence [75]. HDAC6 accumulation in cisplatin-resistant OSCC cells inhibited cell apoptosis by reducing reactive oxygen species (ROS) levels, diminishing DNA damage and increasing recombinant peroxiredoxin 2 (PRDX2) expression [75].

Moving on, extensive research has demonstrated the significant involvement of HDAC10 in various facets of cancer biology, encompassing cell proliferation, apoptosis, metastasis, angiogenesis and drug resistance, albeit with distinct functions contingent upon specific cancer types [76]. Examination of TCGA database indicated upregulated expression of HDAC10 in HNSCC samples. Additional inquiries are imperative to unravel the exact mechanism and contribution of HDAC10 in HNSCC biology.

#### Class III HDACs

Human sirtuin proteins catalyze the deacetylation of histone and non-histone substrates and are involved in cellular localization, protein interaction and enzyme activity regulation. Distinct subcellular localization and substrate specificity define the functional specialization of mammalian sirtuins [77]. SIRT1, SIRT6 and SIRT7 predominantly reside in the nucleus, where they influence chromatin stability and gene transcription. SIRT2 is mainly in the cytoplasm and facilitates deacetylate proteins there. SIRT3, SIRT4 and SIRT5 are predominantly in the mitochondria and control crucial metabolic pathways related to mitochondrial energy production. While SIRT1-3 demonstrates robust sirtuin activity, SIRT4-7 are only detected with very weak activity [78]. Mammalian sirtuins assume diverse functions in cancer, encompassing maintaining genomic stability, regulating metabolism and impacting the tumor microenvironment [79, 80]. Understanding the molecular pathways influenced by sirtuins can help accelerate the development of targeted cancer therapies.

SIRT1 functions as a dual-role factor in HNSCC. SIRT1 expression was significantly decreased in HNSCC compared to normal tissue (*p* < *0.05*) according to TCGA database. SIRT1 expression was significantly higher in the normal epithelial cell line (HaCaT) compared to SCC-9 and SCC-25 cell lines. Overexpressing SIRT1 exerted a robust inhibitory effect on the proliferation and migration of OSCC cells [81]. SIRT1 was bound to the promoter of TGF-β, impeding CBP/p300-mediated acetylation and leading to transcriptional suppression of OSCC [78]. SIRT1 suppressed OSCC by inhibiting vimentin and N-cadherin expression, reducing invasion and migration-related genes (slug, csk2a2, actb, fra1) [82–84]. It also hindered EMT in OSCC by suppressing Smad2/3 phosphorylation and Smad4 deacetylation [84]. Disruption of SIRT1-induced deacetylation of c-JUN by miR-135b-5p promoted migration and invasion in NPC cells [85]. The Kaplan–Meier survival analysis further demonstrated that patients with increased levels of SIRT1 experienced significantly prolonged overall survival, as indicated by Logrank* p* < *0.05* (Fig. 3), suggesting the potential of SIRT1 level as a valuable prognostic biomarker. SIRT1 expression was found to be predominant in cases involving patients aged over 65 years, lymph node negative and early clinical stage cases for HNSCC [86], suggesting its potential as a valuable prognostic assessment tool. The single gene nucleotide polymorphisms (SNPs) in SIRT1 were linked to the survival rate of LSCC patients [85]. Notably, the SIRT1 rs3758391 T/T genotype demonstrated a significant association with an elevated likelihood of developing LSCC [85]. In OSCC, the overexpression of SIRT1 in Tca8113 cell lines led to cisplatin resistance, which could be reversed by BML-210, an inhibitor of class III HDACs [87]. Downregulation of SIRT1 resulted in the inhibition of the PI3K/AKT/mTOR pathway, reversing the radio-resistance of ESCC (ECA-109) cells and improving the prognosis [88]. Within NPC, SIRT1 facilitated the upregulation of SSRP1 expression through catalyzing H3K4 methylation, thereby inducing EBV-miR-BART4 mediated stemness and cisplatin resistance in carcinoma side cells [89]. Moreover, capsaicin hindered SIRT1 activity, thereby augmenting the acetylation of unc-51-like autophagy-activating kinase 1, which subsequently instigated autophagy in OSCC [90]. This implies that SIRT1 may inhibit autophagy in OSCC, although further investigation is needed to comprehensively comprehend this function.

SIRT2, a crucial regulator involved in immune evasion, cell cycle control, energy metabolism and invasion, exhibits noteworthy characteristics in the context of HNSCC [91, 92]. Analysis of the TCGA, CPTAC and HPA databases indicated a significant decrease in SIRT2 mRNA expression in HNSCC compared to normal tissue (*p* < *0.05*). Additionally, Kaplan–Meier survival analysis yielded statistical significance (Logrank *p* < *0.05*), underscoring the potential significance of SIRT2 in validating the prognosis (Fig. 3). SIRT2 has been identified as a key player in the regulation of vascular endothelial growth factor (VEGF) D expression and lymph angiogenesis by deacetylating endothelial PAS domain protein 1 (EPAS1) at Lys674 in HNSCC [93]. Consequently, reagents targeting SIRT2 could potentially yield beneficial outcomes in effectively inhibiting HNSCC lymph angiogenesis [93].

SIRT3 has demonstrated dual functions as a carcinogen or a suppressor in HNSCC. Microarray analysis on OSCC cells, including HSC-3, UM-SCC-1, and UM-SCC-17B, has revealed the overexpression of SIRT3 compared to normal HOKs [94]. However, there was a notable decrease in enzymatic deacetylation in OSCC, indicating the presence of SIRT3 variations [95]. Sequencing of SIRT3 gene in 21 OSCC patients found that 23.8% had the p.Val208Ile mutation [95], which hindered catalytic activity and promoted oral carcinogenesis. SIRT3 downregulation also significantly restrained proliferation and triggered apoptosis of EC9706 cells in ESCC [96]. Notably, the novel SIRT3 inhibitor, LC-0296, exhibited enhanced selectivity toward SIRT3 enzyme, promoting HNSCC cell apoptosis and reducing cell viability when employed alongside radiation or cisplatin therapy [97]. The dysregulation of mitochondrial tumor suppressor genes, including SIRT3, SIRT4 and mitochondrial tumor suppressor 1, was associated with the decrease of mitochondrial DNA repair gene (OGG1-2a) expression, which was important in HNSCC proliferation [98]. SIRT3, a crucial regulator of mitochondrial redox, mitigated the augmented oxidative stress induced by miR-31 in OSCC, thereby promoting tumor cell migration and invasion in OSCC and enhancing the tumorigenic potential of FaDu cells [99, 100]. This could potentially be attributed to the disruption of mitochondrial structure and function in OSCC cells, resulting from perturbations in miR-31 concentration, which subsequently elevated ROS levels and impeded mitochondrial membrane potential [99]. Moreover, suppression of SIRT3 resulted in the inhibition of cellular growth and proliferation, while simultaneously promoting apoptosis in OSCC [94]. This phenomenon was attributed to the elevation of ROS within mitochondria and the augmentation of acetylation levels in mitochondrial proteins, namely NDUFA9 and GDH, consequently inducing mitochondrial fission [94]. In vitro experiments demonstrated that the downregulation of SIRT3 resulted in increased sensitivity of OSCC cells to radiation and cisplatin treatments [101], causing a significant reduction in tumor burden.

SIRT4 has been found with a notable impact on the inhibition of tumor metabolism, specifically in relation to glutamine metabolism [102], indicating a potential anti-tumor effect. Increased expression of SIRT4 in response to DNA damage led to cell cycle arrest and mitigated DNA damage accumulation. Conversely, the downregulation of SIRT4 in non-tumorous cells led to the accumulation of cellular mutations and subsequent tumor formation [103]. The expression of SIRT4 in HNSCC samples appeared lower than in normal ones according to TCGA database. Similarly, Wan et al. [104] investigated a cohort comprising 168 pairs of LSCC tissues, wherein they identified a significant decrease in the expression of SIRT4. This finding suggested a potential association between the downregulation of SIRT4 and the manifestation of a more aggressive tumor phenotype, as well as an unfavorable prognosis [104].

SIRT5 acted as a tumor suppressor by inhibiting the Warburg effect, combating ROS damage and curbing cell proliferation and metastasis [105]. Meanwhile, it could display oncogenic characteristics, leading to chemotherapy and radiotherapy resistance [105]. The bioinformatics analysis revealed a notable upregulation of HDAC3 and SIRT5 expression in OSCC relative to normal tissues, as well as a robust correlation between HDAC3 and SIRT5 expressions and the poor prognosis of OSCC patients [106]. However, TCGA database analysis didn’t indicate a significant difference (*p* > *0.05*) in SIRT5 expression between HNSCC and normal tissues. Further research is still required to unravel the expression patterns and functions of SIRT5 in HNSCC.

Genetic expression profiling of 34 HNSCC patients and TCGA database both revealed a significant upregulation of SIRT6 in the cancer group [107]. Furthermore, the mRNA and protein expression levels of SIRT6 were also found to be higher in OSCC tissues compared to noncancerous tissues [108]. As the direct target of miR-34a, down-regulation of SIRT6 replicated the pro-differentiation effects of miR-34a [109], thus playing a pivotal role in SCCs. What’s more, the expression of SIRT6 was found to be predominantly observed in patients aged 65 years or older, and this association was statistically significant with regards to shorter overall survival [28]. However, there were opposing opinions regarding the anti-tumoral function of SIRT6. A total of 82 cases of OLP and 77 cases of OSCC were examined, revealing that non-dysplastic lesions exhibited significantly higher expression of SIRT6 compared to OSCC lesions [108]. Additionally, in the xenograft mouse model of HNSCC, tumors injected with SIRT6 exhibited significantly reduced volume and weight compared to the control group [110]. MDM2, a negative regulator of the p53 tumor suppressor, played key roles in mediating the anti-tumor effects of SIRT6, as it degraded SIRT6 via the proteasome-dependent pathway, then effectively decreased the proportion of HNSCC cells in the G1 phase, indicative of cell death [110]. SIRT6 overexpression inhibited the NF-κB signaling, reducing the expression of anti-apoptotic protein Bcl-2 while concurrently promoting the expression of pro-apoptotic proteins Bax and caspase-3, ultimately inducing apoptosis in nasopharyngeal carcinoma [111]. SIRT6 also took part in the OSCC senescence by modulating telomere maintenance and DNA repair [110], further underscoring its role in HNSCC.

SIRT7 is a newly discovered regulator of lifespan and senescence with tumor metastasis suppressor properties in OSCC [82]. SIRT7 overexpression effectively inhibited EMT in OSCC by promoting the degradation and deacetylation of Smad4, inducing a cascade of biochemical changes, such as increased E-cadherin, reduced N-cadherin and Vitamin D and reduced matrix metalloproteinase (MMP)-9 [112, 113], consequently, attenuated OSCC invasion, migration and metastasis. Additional research has revealed that miR-770 served as an upstream factor of SIRT7, thereby facilitating the migration and invasion of OSCC cells through the activation of SIRT7/Smad4 signaling pathway [113]. Sripodok et al. [108] observed significantly higher levels of SIRT1 expression in OSCC compared to OLP. Additionally, a positive correlation was found between SIRT7 expression in OSCC and stromal lymphocytic infiltration as well as invasion depth [108]. OSCC cases with elevated SIRT7 expression exhibited a slightly lower probability of survival, although this difference did not reach statistical significance (*p* = *0.1019*) [108]. However, TCGA database did not provide sufficient evidence to support the existence of a significant difference in SIRT7 expression levels between HNSCC and normal tissues. Malik et al. [114] suggested that the effect of SIRT7 on EMT may be tightly regulated by SIRT1 through genetic transcription, an avenue that warrants further investigation and confirmation.

#### Class IV HDAC

Class IV HDAC features a sole member, HDAC11, which is the shortest HDAC protein known and mainly consists of the core catalytic domain. HDAC11 has demonstrated the ability to interact with other proteins, such as HDAC6 and the survival of motor neurons complex, thus engaging in various physiological and pathological processes, including the regulation of mRNA splicing. HDAC11 displays varying expression levels and carries out distinct biological functions in different tissue contexts. In certain cancers, such as hepatocellular carcinoma, breast cancer and renal pelvis urothelial carcinoma, HDAC11 ranked among the top 1–4% of overexpressed genes [115]. Intriguingly, contrary to these cases, the expression of HDAC11 in HNSCC showed a downward trend. Few studies on the function and mechanism of HDAC11 in HNSCC have been carried out and further exploration is urgently needed. Such endeavors hold the potential to unravel novel insights into the involvement of HDAC11 in HNSCC biology.

In conclusion, the employment of unpaired two-tailed t-test for evaluating the mRNA levels of HDAC family genes indicated that a substantial proportion of the genes (13/18, 72.2%) displayed statistically significant differences between HNSCC cases and normal controls. Specifically, HDAC 1, 2, 8, 9, 10 and SIRT 6 displayed higher expression levels in HNSCC cases compared to normal ones. Conversely, the expression of HDAC 4, 5, 6, 11, SIRT 1, 2 and 4 was significantly reduced in HNSCC samples. Protein expression of these HDACs was examined through CPTAC and further confirmed by the HPA database. Protein analysis has the potential to complement RNA data. Specifically, in the context of HNSCC, protein levels of HDAC1, 2 were found to increase, while levels of HDAC4, 5, 6, SIRT 2 decreased. Levels of HDAC7 and SIRT7 proteins did not exhibit a significant difference between HNSCC and normal samples. The Kaplan–Meier plotter highlighted the prognostic importance of HDAC mRNA expressions in HNSCC patients. Survival analysis showed significantly longer overall survival for patients with elevated levels of SIRT1 and SIRT2 (Logrank* p* < *0.05*), suggesting their value as prognostic biomarkers.

#### Predicting functions and signals of HDACs and neighboring genes in HNSCC

We analyzed a set of 50 neighboring genes that exhibited significant associations with HDACs utilizing the GeneMANIA database. The five genes most closely associated with HDACs were AL133500.1, DHPS, AGMAT, ARG2 and ARG1. As shown in Fig. 4A, 89.03% of these genes had shared protein domains, 5.70% shared physical interactions, 1.94% shared pathway, 1.63% shared prediction, 1.21% of these genes shared co-expression and only 0.49% shared co-localization. A protein interaction network was constructed by online software (STRING) and core factors were further screened from the network. The results showed that most of these factors were positively correlated and the PPI network revealed HDAC1, HDAC2 and SIRT2 as top3 hub proteins (Fig. 4B). Among them, HDAC1 and HDAC2 emerged as highly related proteins, boasting an astounding 85% global sequence identity [116]. Delving beyond their structural similarities, these proteins exhibited functional redundancy across a plethora of biological processes. Intriguingly, when HDAC1 and HDAC2 were jointly deleted, a remarkable escalation in impact ensued, inducing profound disruptions in mitosis and instigating cellular demise [117, 118], highlighting the untapped potential for synergistic effects arising from the concomitant abrogation of these HDACs. Additionally, we performed GO enrichment and KEGG pathway analyses on the HDACs and the aforementioned 50 neighboring genes using OmicShare. From this analysis, we identified the top 30 most highly enriched GO items. The findings from the GO term analysis indicated that the genes exhibiting differential expression primarily served as deacetylase activity. Furthermore, the cellular components predominantly encompassed the histone deacetylase complex, nucleoplasm, transcriptional repressor complex, membrane-enclosed lumen and organelle lumen. Additionally, the biological processes primarily involved protein deacetylation, protein deacylation, macromolecule deacylation, histone H3 deacetylation and histone deacetylation. The KEGG pathway analysis indicated that the differentially expressed genes were predominantly linked to alcoholism, viral carcinogenesis, nicotinate and nicotinamide metabolism, microRNAs in cancer, the longevity regulating pathway, among others.

## HDAC inhibitors in HNSCC treatment

Combining surgical intervention with radiotherapy and/or chemotherapy, such as cisplatin and 5-fluorouracil, is a recommended approach for the treatment of HNSCC. However, the non-specific cytotoxicity of these interventions poses limitations to their efficacy and increases the risk of adverse effects [119]. In recent years, several inhibitors targeting HDAC activity have shown promise and progressed to clinical trials. Notably, vorinostat (SAHA) was the first HDAC inhibitor to receive approval from the U.S. Food and Drug Administration (FDA) for the treatment of cutaneous T-cell lymphoma (CTLC) [120]. Subsequently, romidepsin (FK228) received FDA approval for the treatment of CTLC and peripheral T-cell lymphoma (PTL) in 2009 and 2012 respectively [121, 122]. In 2014, Belinostat (PXD101) was approved by the FDA for the treatment of PTL [123]. Additionally, the FDA has approved sodium phenylbutyrate (4-PB) for the treatment of urea cycle disorders [124]. Furthermore, a novel HDAC inhibitor, CG-745, has been granted Orphan Drug Designation by the FDA for the treatment of pancreatic cancer and is presently undergoing Phase II clinical trials (http://www.crystalgenomics.com/). These significant advancements underscore the necessity and rationale for incorporating a growing array of HDAC inhibitors into clinical trials.

In general, effective HDAC inhibitors are composed of three distinct components: the surface recognition part, also referred to as CAP, which obstructs access to the active site; the zinc binding group (ZBG), which forms chelation with the active site; and the linker that connects these two regions [125]. While hydroxamates are commonly utilized as HDAC inhibitors, other zinc-binding groups such as benzamides, sulfonamides, thiols and ketones are also employed to enhance the specificity of inhibitors. The surface recognition part has the ability to bind to the HDAC itself, as well as other complexes near the active site, making it a valuable tool for designing inhibitors that target specific HDAC [126]. Linker modifications can also improve specificity, for example, by adding aromatic rings [127]. Table 2 provides a summary of the HDAC inhibitors that have been tested for their efficacy in HNSCC treatment, along with their respective underlying mechanisms.

**Table 2:** HDAC inhibitors in HNSCC treatment

Classification	Laboratory code	HDAC target	Therapeutic mechanisms	Clinical trials
Hydroxamic acids	Anilide hydroxime (Vorinostat, SAHA)	Class I, II, IV	Inhibiting cell proliferation, reversing EMT, inhibiting migration and invasion [131]	Vorinostat with cisplatin/RT in phase I trial for locally advanced HNSCC had a 96% complete response rate and a 5-year overall survival rate of 68.45% [195] Vorinostat with pembrolizumab in phase II trial for HNSCC and salivary gland cancer (R/M) revealed a 32% partial response [201]
	Trichostatin A (TSA)	Class I, II	Producing growth arrest, arresting cell cycle, proapoptotic and radio-sensitization [132]	
	LBH589 (Panobinostat)	Class I, II, IV	Producing growth arrest, arresting cell cycle [141]	Panobinostat combined with erlotinib in phase I trial for HNSCC had good tolerance, with 3/7 HNSCC patients achieved stable disease and a 43% disease control rate [200]
	Quisinostat (JNJ-26481585)	HDAC1	Inhibiting proliferation, antimigration, inducing cell apoptosis, cell death and cell pyroptosis [143]	
	Scriptaid	HDAC1, 3, 8	Inhibiting DNA damage repair, increasing radiosensitivity [143]	
	A1659	HDAC1, 6	Producing growth arrest, arresting cell cycle, proapoptotic [146]	
	Rocilinostat (ACY1215)	HDAC6	Inhibiting proliferation, promoting programmed cell death, apoptosis and necro-like cell death [147, 149]	
	6 h		Inhibiting cell proliferation [151]	
	3c, 3d		Inhibiting cell proliferation, inducing apoptosis [152]	
Cyclic tetrapeptides	Peptide-FK228 (Romidepsin)	HDAC1, 2	Arresting cell cycle, increasing radio-sensitization [185]	Romidepsin in phase II trial for HNSCC revealed pharmacodynamic effects [154]
	Apicidin	HDAC2, 3	Producing antiproliferative, proapoptotic and promoting autophagy effects [52]	
	Largazole	Class I	Producing antiproliferative [160]	
	16c	HDAC1, 6	Producing antiproliferative [160]	
	4j		Promoting autophagy, reversing the cisplatin resistance in Cal27CisR [161]	
	H1, H2, I2, J2, K1, K2		Inhibiting cell proliferation, increasing radiosensitivity [162]	
Benzamides	Entinostat (SNDX-275, MS-275)	HDAC1, 9, 11	Arresting cell cycle, producing proapoptotic effect [168]	
	Tucidinostat (chidamide)	HDAC1, 2, 3, 10	Inducing apoptosis, pyroptosis and ferroptosis [169]	
	Mocetinostat (MGCD0103)	Class I	Increasing radio-sensitization, increasing cell cycle arrest [170]	
	Domatinostat (4SC-202)	HDAC1, 2, 3	Inhibiting cell growth, reducing EMT, decreasing invasion and migration, preventing recurrence	
Aliphatic fatty acids	Valproic acid (VPA)	Class I	Producing DNA damage, antiproliferative and proapoptotic effects [179]	VPA with cisplatin/RT in phase II trial for locally advanced HNSCC was terminated early due to toxicities [196]
	Sodium butyrate (NaB)	Class I, IIa	Controversial effects on cell proliferation and invasion [185]	
	(S)-HDAC42	Class I, II	Producing proapoptotic effect [185]	
Others	NDACI054	Class I, II	Inhibiting cell proliferation, increasing radiosensitivity [187]	
	A248	HDAC1, 6	Producing growth arrest, arresting cell cycle, proapoptotic [187]	
	LMK235	Class I, IIb	Increasing chemo-sensitization [188, 189]	
	13a, 13d		Increasing chemo-sensitization [190]	
	EGCG	SIRT3	Suppressing SIRT3 in oral cancer cells while stimulating SIRT3 in normal cells [100]	
	LC-0296	SIRT3	Elevating ROS levels, impeding cell viability, inducing apoptosis [94]	
	CUDC-101	Class I, II	Antiproliferative and proapoptotic activities [192]	CUDC-101 with cisplatin/RT in phase I trial for intermediate/high-risk HNSCC showed a 75% complete response rate but a high rate of dose-limiting toxicity-independent discontinuation [191]

### Hydroxamic acids

The development of hydroxamic acid HDACIs was built upon the foundation of dimethyl sulfoxide (DMSO). In their investigation of mouse erythroleukemia cell resuscitation, Friend et al. [128] made a notable discovery—the inhibitory effect of DMSO on the growth of passing cells, resulting in significant improvement in two-thirds of the diseased cells. This intriguing phenomenon captured the attention of Marks et al. [129] and marked the beginning of extensive research into the potential of hydroxamic acids HDACIs. At the moment, hydroxamic acid is the most widely utilized ZBG in HDAC inhibitors, with several marketed or clinically tested inhibitors incorporating hydroxamic acid, including trichostatin A (TSA), anilide hydroxime (SAHA), belinostat (PDX101), panobinostat (LBH589), quisinostat (JNJ-26481585), among others [130]. The chelation of the carbonyl and hydroxyl groups of hydroxamic acid with zinc ions within the HDAC binding site determines the inhibitory activity of HDAC inhibitors. Moreover, the hydrophobic chain of hydroxamic acid enables interaction with the hydrophobic channel of HDAC, facilitating the approach and interaction of the ZBG with zinc ions [30].

SAHA, a non-selective HDAC inhibitor, is the first FDA-approved HDAC inhibitor for single-agent or combination therapy in CTLC. SAHA induced hyperacetylation of histones H2A and H3, leading to reduced cell viability and inhibition of anchorage-independent growth in HSC-3 and HSC-4 cells. DAPI staining and WB analysis have demonstrated that SAHA induced caspase-dependent apoptosis in HSC-3 and HSC-4 cells. When combined with cisplatin or gefitinib, SAHA inhibited the proliferation, migration and invasion of HNSCC cells and reversed the EMT process [131].

The anti-proliferative effects of TSA were observed in HNSCC through the downregulation of tight junction molecules mediated by p63, as well as the induction of growth arrest mediated by either p63 or p21 [132]. TSA acted on cyclins in different cell cycle phases, delaying G1/S transition via cyclin D1 [133], preventing S/G2 transition via cyclin A [132] and delaying G2/M transition via cyclin B [134]. TSA activated endogenous apoptosis by inducing expression of apoptotic proteins (e.g., BAX, BAK, BID), inhibiting expression of anti-apoptotic proteins (e.g., SURVIVIN) [135] and reducing mitochondrial membrane potential or lysosomal pH [136]. Moreover, TSA down-regulated the expression of pro-angiogenic genes (e.g., VEGF), thus inhibiting angiogenesis and improving overall survival. Additionally, TSA enhanced the sensitivity of OSCC cell lines to ionizing radiation, promoted radiation-induced apoptosis in TSCC cells and reversed acquired radio-resistance, presenting a potentially promising approach for TSCC treatment [137].

The HDAC inhibitor Panobinostat (LBH589) has demonstrated its efficacy in blocking multiple cancer-related pathways and reversing epigenetic changes associated with cancer [138]. LBH589 is known for its pan-deacetylase inhibitory activity against Class I, II and IV HDACs [138]. Numerous studies have demonstrated that LBH589 effectively suppresses various hematological malignancies, including lymphoma, multiple myeloma and acute myeloid leukemia, at concentrations as low as nanomolar levels [139, 140]. In the context of OSCC, LBH589 has been observed to enhance the expression of p27 and p21, while reducing the expression of cyclin D1 and myeloid leukemia-1 (MCL-1). Additionally, LBH589 significantly impeded cell growth and diminished the sub-G1 cell population. LBH589 activated Sp1 to induce apoptosis in OSCC cells through changes in the expression of BAX, BID and BCL-xL [141].

Quisinostat (JNJ-26481585) exhibited a broad-spectrum antiproliferative effect on various cancer cells, including breast, lung, colon and prostate cancer cells, at low nanomolar concentrations (30–100 nM) [142]. In TSCC, quisinostat inhibited cell proliferation and migration, induced tumor cell apoptosis, altered the expression of caspase-1 protein and triggered pyroptosis. Furthermore, quisinostat increased ROS levels in TSCC cells, reduced the expression of recombinant glutathione peroxidase 4 (GPX4) and induced TSCC cell death through ferroptosis-related pathways [143].

The efficacy of Scriptaid in augmenting the radiosensitivity of SQ-20B human laryngeal squamous cell carcinoma cells through the inhibition of DNA damage repair has been demonstrated [144]. In an endeavor to enhance HDAC inhibition and metabolic stability, Professor Gyoonhee Han synthesized A1659 [145]. This compound exhibited the ability to suppress the expression and nuclear translocation of Sp1, regulate the expression of p27 and cyclin D1, induce apoptosis and markedly decrease the viability of MC-3 and HN22 human oral cancer cell lines [146].

Rocilinostat (ACY1215), a specific inhibitor of HDAC6, has demonstrated efficacy in inhibiting tumor growth when used alone or in combination with other medications in various types of cancer [147, 148]. Recent research has revealed that ACY-1215 hindered cell proliferation and promoted programmed cell death in ESCC through the involvement of the ERK as well as the miR-30d/PI3K/AKT/mTOR pathways [147, 149]. Additionally, the concurrent administration of ACY1215 and adavosertib in HNSCC cells has been found to suppress Chk1 activity, leading to a synergistic enhancement of apoptosis through mitotic catastrophe [149]. The co-administration of proteasome inhibitor Bortezomib (BTZ) and ACY1215 to CAL27 and Detroit 562 HNSCC cells resulted in the imbalance of ROS and induced necro-like cell death [150], thus demonstrating potential as a therapeutic approach for HNSCC.

Sun et al. [151] designed and synthesized a hydroxamic acid-based HDAC inhibitor, referred to as 6 h (Additional file 1: Fig. S1), utilizing 4,5,6,7-tetrahydrobenzothiazole as the structural core. The antiproliferative activity of 6 h in KYSE30 human ESCC cells was assessed through MTT bioassay, employing ACY1215 as reference standards. The results indicated that 6 h displayed significantly superior antiproliferative activity compared to ACY1215 in KYSE30 cells, as evidenced by IC_50_ values of 4.19 µM and 19.84 µM, respectively [151], suggesting that further investigation into the potential application of 6 h in cancer treatment is warranted.

Potential dual mode anticancer agents were synthesized through combine the antivascular effect of the 4,5-diarylimidazole moiety with HDAC inhibition by the (4-aryl-1-methylimidazol-5-yl) cinnamoyl hydroxamate [152]. The efficacy of the structures of new imidazoles with hydroxamic acid appendages (3c, 3d) (Additional file 1: Fig. S1) was evaluated on Kyse-140 ESCC cells, resulting in growth inhibition and induced apoptosis, which was demonstrated through MTT assays and an observed increase in caspase-3 activity [152].

### Cyclic peptides

Cyclic peptides are among the most intricate compounds of all inhibitors, featuring large rings comprising amino acids in CAP, functional groups in ZBG and alkyl chains in the linking domain. The inclusion of the macrocyclic surface recognition structure within the inhibitor complex not only confers structural stability, but also facilitates a more extensive interaction between the inhibitor and enzyme molecules. This pivotal characteristic likely contributes to the robust activity of cyclic peptide inhibitors and serves as a crucial factor in maintaining their efficacy. In recent years, substantial advancements have been made in the advancement of cyclic peptide HDAC inhibitors, primarily owing to their remarkable activity, specificity and minimal cytotoxicity [153].

One notable HDAC inhibitor is Romidepsin (FK228), which is the second FDA-approved inhibitor for CTCL therapy and the first phase II clinical trial HDAC inhibitor targeting HNSCC. A Phase II clinical trial (NCT00084682) was conducted involving 14 patients with advanced HNSCC, where tumor, blood and uninvolved oral mucosa samples were collected for analysis before and after treatment with Romidepsin as a single agent [154]. The trial showed Romidepsin had pharmacodynamic effects but no objective responses [154]. Tolerability is a concern, but combining HDAC inhibitors with other therapies may be promising [154]. Analysis of the samples using microarray revealed 641 differentially expressed genes following Romidepsin treatment, which was commonly linked to transcriptional regulation, cell cycle control, signal transduction and electron transport [154]. The combination of Romidepsin with adenoviral gene therapy targeting p53 in esophageal Tn and TE2 SCC cell lines demonstrated heightened efficacy, as evidenced by increased radiosensitivity and induction of cell cycle arrest [155]. Meanwhile, Romidepsin was found to enhance the proapoptotic effects of Ad-p63 and Ad-p73 [155]. While Romidepsin alone effectively inhibited HDAC in HNSCC, its effectiveness was constrained, leading to its predominant use in conjunction with other HDAC inhibitors.

Another compound that shows promise is Apicidin, which is a fungal metabolite derived from Fusarium fermentation [156]. It has been found to selectively reduce HDAC8 expression in OSCC AT-84 cells, leading to significant growth inhibition. In an experimental murine tumor model, the administration of Apicidin resulted in a notable reduction of 14% in tumor size when compared to the control groups following a 46-day treatment period. Subsequent immunohistochemistry analysis indicated that Apicidin effectively hindered the proliferation of OSCC cells, triggered caspase-dependent apoptosis and facilitated autophagy. These observed effects were attributed to the heightened expression levels of p21^WAF1/Cip1^ and the induction of G2/M cell cycle arrest [52].

In 2008, Leusch et al. [157, 158] discovered Largazole, a potent and selective Class I HDAC inhibitor. Largazole fluorination displayed favorable tolerance towards HDAC inhibitory activity and selectivity [159]. Further modification of the valine residue within the macrocyclic moiety of fluoro-largazole using S-Me l-Cysteine to synthesize compound 16c (Additional file 1: Fig. S1) exhibited a substantial increase in the inhibition of HDACs, ranging from 5 to ninefold, while preserving the selectivity towards HDAC1, 6 [160]. Additionally, this modified analog demonstrated significant growth inhibition against ECA-109 cells, displaying potency levels comparable to those of largazole [160].

The synthesis conducted by Krieger et al. [161] involved the hydroxamate HDACi 4j (Additional file 1: Fig. S1), which exhibited impressive chemo-sensitizing properties. The application of 4j greatly augmented the sensitivity of the cisplatin-resistant subline Cal27CisR to cisplatin, leading to an approximate sevenfold amplification [161]. Moreover, 4j effectively reversed the cisplatin resistance in Cal27CisR, primarily through the activation of apoptosis [161].

Jung et al. [162] orchestrated the design and screening of more than 60 analogues of HDAC inhibitors, encompassing a urea backbone and the hydroxamic acid end moiety. Notably, six of these analogues (H1, H2, I2, J2, K1, K2) (Additional file 1: Fig. S1) exhibited a significant 50% reduction in HDAC enzyme activity at nanomolar concentrations [162]. The IC_50_ values for inhibiting cell proliferation varied from 10 to 50 microM across different cancer cell lines, including SQ-20B HNSCC cells [162]. Furthermore, the compounds demonstrated remarkable radio-sensitizing properties as evidenced by radiation clonogenic survival assays [162].

### Benzamides

For over a decade, researchers have conducted investigations on benzamides as potential zinc-binding groups for HDAC inhibitors [163]. In comparison to hydroxamic acids, HDAC inhibitors based on benzamides offer greater selectivity and improved safety for class I HDACs. Tucidinostat/chidamide received approval from the former China Food and Drug Administration (CFDA) in 2014 for the treatment of HR+/HER2− breast cancer and PTL. Currently, entinostat/MS-275, mocetinostat/MGCD0103 and domatinostat/4SC-202 are undergoing phase II clinical studies [30]. The compound CI-994, which demonstrates inhibitory activity on HDAC and effectively induces G1/S phase arrest, was obtained by acetylating dinaline, a traditional anticonvulsant with inhibitory effects on cell growth [164]. With its broad-spectrum anti-tumor activity, CI-994 has emerged as the first benzoamide HDAC inhibitor to undergo clinical trials. Currently, CI-994 is being evaluated in phase II clinical trials in combination with gemcitabine for the treatment of solid tumors, including non-small cell lung cancer and colon cancer [165].

Entinostat (SNDX-275 and MS-275), a bioactive inhibitor of Class I HDAC with an extended half-life, is the first synthetic small-molecule benzamide derivative being tested in multiple clinical trials [166, 167]. The compound has demonstrated the ability to enhance acetylation modifications of histone H3 and H4, leading to a dose-dependent reduction in viability of OSCC cells. Entinostat has been found to induce cell cycle arrest in the G0/G1 phase and inhibit the proliferation of OSCC cell lines by modulating genes associated with the cell cycle, such as p21. Furthermore, entinostat has been shown to facilitate ROS-induced apoptosis by regulating the expression of thioredoxin binding protein 2 (TBP2), thioredoxin (TRX) and other factors crucial for maintaining tissue homeostasis [168]. Although entinostat is currently being evaluated in clinical trials, such as a multicenter Phase II study initiated in 2017 to evaluate its effectiveness in advanced hormone-resistant or triple-negative breast cancer [166], additional research is necessary to evaluate its potential application in HNSCC.

Tucidinostat/chidamide, a selective inhibitor of class I and IIb HDACs (1, 2, 3 and 10), was known to demonstrate anti-cancer properties against TU212 and AMC-HN-8 cells, resulting in diverse forms of cell death in LSCC [169]. Chidamide effectively induced apoptosis, pyroptosis and ferroptosis through the activation of caspase-3, caspase-1 and Gpx4 [169], respectively, suggesting a promising therapeutic approach for HNSCC.

When HNSCC cells were exposed to a combination of 5-aza-20-deoxycytidine (DAC) and HDAC inhibitors such as LBH589 or mocetinostat (MGCD0103), it led to increased sensitivity to radiation therapy. These interventions resulted in histone hyperacetylation, reversal of gene silencing and enhanced cell cycle arrest in response to radiation, although the precise underlying mechanisms require further exploration [170].

The combination of domatinostat (4SC-202), a type I HDAC inhibitor, with metformin has been shown to effectively inhibit the growth of OSCC cells and induce apoptosis by targeting ΔNp63 degradation through ubiquitin [171]. This combination also decreased OSCC invasion and migration by inhibiting TWIST1 expression and STAT3 phosphorylation [172]. Combining 4SC-202 and mTOR inhibitor Ink-128 reduced EMT in OSCC cells by activating FoxO1 and inhibiting Twist1 [173]. Moreover, the combined administration of 4SC-202 and INK128, a specific mTORC1/C2 inhibitor, induced the reduction of SOX2 expression via miR-429/miR-1181 mediated mRNA degradation and inhibition of cap-dependent mRNA translation. This combination significantly impeded oncogenic processes and recurrence in OSCC [174].

### Aliphatic fatty acids

Aliphatic fatty acid inhibitors, whose metal-binding region is found in the clostridium group, require a millimol concentration to produce action with relatively weak overall inhibitory activity, but their market potential cannot be ignored. Presently, the sodium valproate and prednisone combination is undergoing phase III clinical trials for the treatment of B cell lymphoma [175]. Although sodium phenylbutyrate has been launched since 1996, further clinical investigations are imperative to explore its potential as an anticancer agent [176].

Valproic acid (2-valproic, VPA) is a short-chain fatty acid with HDAC inhibitory activity, which has been employed for over five decades in the management of epilepsy, schizophrenia and bipolar disorder [177]. Numerous preclinical investigations, both in vivo and in vitro, have demonstrated the substantial ability of VPA to impede the proliferation of cancer cells through the modulation of diverse signaling pathways. VPA was found to induce considerable DNA damage when combined with cisplatin and cetuximab in HNSCC cells. The limited effectiveness of cetuximab (CX) and cisplatin (CDDP) in HNSCC was attributed to drug resistance and toxicity [178], while VPA played a role in enhancing CX and CDDP functions through targeting the cancer stem cell compartment in particular. VPA was observed to impede the DNA repair process and enhance the intracellular concentration of CDDP through the influx and efflux mechanisms. It also interfered with the activation of survival pathways and DNA repair mechanisms through the induction of epidermal growth factor receptor (EGFR) expression and inhibition of EGFR nuclear translocation, resulting in impaired cyclin D1 transcription and the generation of antiproliferative and proapoptotic effects [179]. The co-administration of VPA and CDDP/CX exhibited a significant reduction in tumor growth, thereby reducing the required drug dosage and associated toxicity, thereby showcasing a promising potential for future applications.

Sodium butyrate (NaB) is another aliphatic fatty acid with HDAC inhibitor activity targeting classes I and IIa members. In OSCC cells, NaB induced G0/G1 arrest and inhibited cell proliferation and invasion by downregulating HDAC1 expression and upregulating HSPB7 levels [180]. Although NaB partially reversed the EMT process [181], it significantly upregulated the expression levels of MMP-1, 2, 9 and 13, thereby enhancing cell migration through the induction of Vimentin- and SNAI1-mediated EMT [182]. In addition, the concentration of butyrate in the saliva of OSCC patients was higher than in control samples, indicating that butyrate could potentially serve as a biomarker for OSCC [183]. The metabolomic analysis of OSCC tissues revealed a positive correlation between elevated levels of butyrate and advanced tumor staging as well as lymph node metastases [184]. These contradictory findings suggested that the specific function of NaB in OSCC remains unclear and further research is urgently needed.

(S)-HDAC42, a compound that targets classes I and II members, has been found to possess significant antitumor activity against OSCC cell lines including Ca922, SAS and HSC-3. The compound effectively down-regulated phosphorylated Akt and cell cycle-related proteins such as cyclin D1 and CDK6, mediating caspase-dependent apoptosis. Additionally, (S)-HDAC42 inhibited the NF-κB pathway by interfering with the nuclear translocation induced by tumor necrosis factors and the production of active reactive oxygen species, resulting in the growth inhibition of OSCC cell lines [185]. Compared to SAHA, (S)-HDAC42 exhibited a reduced IC_50_ in its ability to suppress tumor cells, showing promising efficacy in the mouse model [186].

### Other HDACIs

Novel HDAC inhibitors have recently emerged as potential therapeutic agents in the context of HNSCC. Significant discoveries have been made by researchers, revealing compounds that show promising inhibitory effects on HDAC activity. One such compound is NDACI054, a novel inhibitor that targets both class I and class II HDACs. In an experimental setting using UT-SCC15 HNSCC cells cultured in both two-dimensional and three-dimensional conditions, NDACI054 exhibited remarkable inhibitory effects on cell survival at low doses ranging from 2.5 to 5 nM. NDACI054 also demonstrated the ability to enhance the radiosensitivity of UT-SCC15 cells [187]. These findings highlight the potential of NDACI054 as a therapeutic agent for HNSCC, particularly in combination with radiotherapy. Another intriguing HDAC inhibitor is A248, a pyridine-based HDAC inhibitor, it could attenuate the expression and nuclear translocation of Sp1, which was involved in the regulation of important cell cycle proteins such as p27 and cyclin D1. Through the modulation of these proteins, A248 could induce apoptosis and significantly reduce the viability of MC-3 and HN22 cells [146]. The manipulation of the CAP and linker region of vorinostat resulted in the creation of alkoxyamide based compound LMK235, a potent HDAC inhibitor exhibiting preferences for class I and class IIb HDACs and demonstrated the ability to sensitize HNSCC cells to chemotherapy [188, 189]. In order to augment the anticancer efficacy, the incorporation of tri- and dimethoxy-phenyl substitutions is proposed within the structure of LMK235 to form the compounds 13a and 13d (Additional file 1: Fig. S1) [190]. When co-administered with cisplatin, both 13a and 13d exhibited enhancement of the cytotoxic effects caused by cisplatin, primarily through the activation of the caspase 3 and caspase 7 pathway in Cal27CisR cells [190]. Pretreatment with 13d fully reinstated the sensitivity of Cal27CisR cells to cisplatin [190], which underscores the potential of 13d as an epigenetic tool for investigating and manipulating cisplatin resistance in HNSCC. Tao et al. [100] demonstrated that the green tea catechin (−)-epigallocatechin-3-gallate (EGCG) exhibited potential as a medicinal agent due to its ability to suppress SIRT3 in oral cancer cells while stimulating SIRT3 in normal cells. Additionally, the novel SIRT3 inhibitor LC-0296 was found to impede cell viability and induce apoptosis by elevating levels of ROS in HNSCC cells [94]. CUDC-101, a compound with multiple pharmacophores, demonstrates inhibitory effects on HDAC, EGFR and HER2 [191]. It showed strong antiproliferative and proapoptotic effects in drug-resistant HNSCC models by inactivating EGFR, HER2 and other survival pathways [192].

### Combination therapy

The incorporation of epigenetic drugs into combination therapies has emerged as a compelling option for the treatment of cancer [193]. By employing rational combination regimens, the limitations of standalone epigenetic therapies can be overcome, leading to improved antitumor efficacy and decreased likelihood of drug resistance. HDAC inhibitors not only enhance tumor sensitivity to radiotherapy, but serve as a safeguard for normal tissues by augmenting their maximum tolerance to radiotherapy [194]. Compounds like TSA [132], romidepsin [185] and mocetinostat [170] can increase the effectiveness of radiotherapy. In the Phase I trial for locally advanced HNSCC, the combination of vorinostat with cisplatin/RT demonstrated a 96% complete response rate and an estimated 5-year overall survival rate of 68.45% [195]. Additionally, in a Phase I trial (NCT01384799) for intermediate/high-risk HNSCC, the combination of CUDC-101 with cisplatin/RT yielded a 75% complete response rate, but was associated with a high incidence of dose-limiting toxicity-independent discontinuation [191]. However, the phase II trial (NCT01695122) evaluating VPA with cisplatin/RT for locally advanced HNSCC was prematurely halted due to significant toxicities [196]. Coupling epigenetic drugs with chemotherapeutic agents that cause DNA damage has emerged as an attractive strategy to prevent or defeat drug resistance. For instance, coupling HDAC inhibitors and DNA methyltransferase (DNMT) inhibitors have been found to have synergistic effects on multiple fronts, including tumor suppressor gene reactivation, apoptosis induction and cell division/growth inhibition in cancer cells [197]. Luan César Silva et al. [198] presented compelling evidence supporting the superior efficacy of a low-concentration treatment strategy utilizing the NF-kB inhibitor emetine in conjunction with SAHA for disrupting cancer stem cells in mucoepidermoid carcinomas.

Current studies target epigenetic approaches to regulate immunity against tumors. Iwasa et al. [199] uncovered resistance mechanisms in OSCC following nivolumab therapy utilizing spatial transcriptomics, with initial tumor pathways linked to immune activities including antigen processing, interferon-gamma signaling and innate immunity. Post-immunotherapy, activation involved epigenetic modifications such as deacetylation by HDAC. Following the emergence of acquired resistance, the activation of pathways associated with epigenetic changes suggested that such alterations in HNSCC could suppress the immune response through the anti-PD-1 antibody. Active testing is underway for epigenetic targeting therapies to enhance anti-tumor immune responses. The combination of panobinostat and erlotinib in a phase I trial (NCT00738751) for patients with HNSCC demonstrated favorable tolerability, resulting in stable disease in 3 out of 7 HNSCC patients and a disease control rate of 43% [200]. Phase II trial (NCT02538510) combining vorinostat with pembrolizumab in patients with HNSCC and R/M salivary gland cancer showed a 32% overall response rate, but had higher adverse events compared to pembrolizumab alone [201]. Another ongoing clinical trial (NCT03019003) is investigating the safety and potential improvement in outcomes of adding azacitidine to the durvalumab/tremelimumab mix for R/M HNSCCs who did not respond to anti-PD-1, anti-PD-L1 or anti-CTLA-4 treatments. Further exploration is urgently needed due to limitations in cohort size and heterogeneity.

The persistence of cancer recurrence following surgery presents a significant challenge, with bone marrow-derived myeloid cells being instrumental in creating the premetastatic environment necessary for tumor dissemination [202, 203]. Treatment after esophageal cancer surgery with low doses of DNA methyltransferase and histone deacetylase inhibitors, such as 5-azacytidine and entinostat, induced the differentiation of myeloid-derived suppressor cells into a macrophage-like phenotype [204]. This disruption of the premetastatic microenvironment inhibited cancer recurrence and lung metastases [204]. Moreover, the combination of 4SC-202 and INK128 reduced SOX2 expression through miR-429/miR-1181 and inhibited cap-dependent mRNA translation, leading to decreased oncogenic processes and recurrence in OSCC [174].

## HDAC agonists in HNSCC treatment

Significant strides have been made in the development and design of HDAC agonists, with a primary focus on targeting the sirtuin family. Among these, the most extensively studied class of sirtuin agonists is known as SIRT1-activating compounds (STACs), with resveratrol being a prominent example [205]. Resveratrol has demonstrated its ability to significantly and specifically increase the affinity of SIRT1 for its substrate small peptides and to effectively promote the deacetylase activity of SIRT1 [206]. Curcumin (diferuloylmethane), a polyphenol derived from Curcuma longa, has shown potential therapeutic properties in the treatment of HNSCC by stimulating SIRT1 [32]. The utilization of curcumin on FaDu and Cal27 cells led to the suppression of cell proliferation, migration and associated angiogenesis, accomplished by activating both the intrinsic apoptotic pathway (caspase 9) and the extrinsic apoptotic pathway (caspase 8) [32]. Additionally, the anticancer characteristics of curcumin can be attributed to the activation of ATM/CHK signal and the inhibition of NF-κB [32]. CAY1059, an additional activator of SIRT1, exhibits inhibitory effects on both cellular proliferation and migratory behavior in Ca9-22 cells of gingival squamous cell carcinoma [83]. Subsequently, Sirtris Pharmaceuticals has developed several novel SIRT1 agonists, such as SRT2183, SRT1460 and SRT1720 [207], with enhanced SIRT1 activation ability. However, these agonists have generated much controversy and doubt and there is a pressing need to find more powerful activating molecules.

## Conclusions and prospect

The emergence and progression of HNSCC are strongly linked to histone acetylation, as described in Fig. 2. The biological functions of histone deacetylase in tumor initiation, promotion and progression are intricate and may be influenced by their tissue- and cancer-specific expression, as well as experimental conditions. Nevertheless, HDAC inhibitors, such as SAHA and Romidepsin, which have been approved for CTCL treatment, hold potential in the treatment of HNSCC.

Researches have shown that certain HDACs can act as oncogenes in the context of HNSCC, HDACs and their inhibitors can affect HNSCC by inducing growth arrest, apoptosis, and autophagy, among others. However, the precise oncogenic roles of some HDAC members in HNSCC are still obscure. In order to ascertain the potential therapeutic advantages of HDAC activation (HDAC agonists) or inhibition (HDAC inhibitors) and their associated adverse effects, it is imperative to acquire a comprehensive understanding of HDACs biology encompassing both the molecular and physiological dimensions.

### Supplementary Information

**Additional file 1: Figure S1.** The molecular structures of novel HDAC inhibitors.

## Data Availability

The data supporting this review are from previously reported studies and, which have been cited.

## Abbreviations

CFDA: China Food and Drug Administration
CoREST: Corepressor of RE1-silencing transcription factor
CRT: Chemoradiation therapy
DMSO: Dimethyl sulfoxide
DNMT: DNA methyltransferase
EBV: Epstein Barr virus
EGFR: Epidermal growth factor receptor
EMT: Epithelial–mesenchymal transition
ER: Endoplasmic reticulum
ESCC: Esophageal squamous cell carcinoma
FDA: The U.S. Food and Drug Administration
GO: Gene ontology
GPX4: Glutathione peroxidase 4
HATs: Histone acetyltransferases
HDACs: Histone deacetylases
HNSCC: Head and neck squamous cell carcinoma
HOKs: Human oral keratinocytes
IHC: Immunohistochemical
KEGG: Kyoto Encyclopedia of Genes and Genomes
LSCC: Laryngeal squamous cell carcinoma
MEC: Mucoepidermoid carcinoma
MEF2D: Myocyte enhancer factor 2D
MMP: Matrix metalloproteinase
nM: Nanomolar
NPC: Nasopharyngeal carcinoma
NuRD: Nucleosome remodeling deacetylase
OA: Overall survival
OED: Oral epithelial dysplasia
OSCC: Oral squamous cell carcinoma
PCNA: Proliferating cell nuclear antigen
PKM2: Pyruvate kinase isozyme type M2
PRDX2: Recombinant peroxiredoxin 2
PTL: Peripheral T-cell lymphoma
RAR: Retinoic acid receptor
ROS: Reactive oxygen species
RT: Radiotherapy
SNPs: Single gene nucleotide polymorphisms
TCGA: The Cancer Genome Atlas
TSCC: Tongue squamous cell carcinoma
VEGF: Vascular endothelial growth factor
WB: Western blot
ZBG: Zinc binding group

## References

[1] Cramer JD, Burtness B, Ferris RL (2019). Immunotherapy for head and neck cancer: recent advances and future directions. Oral Oncol.

[2] Shao B (2023). Molecular evolutionary landscape of the immune microenvironment of head and neck cancer. Biomolecules.

[3] Elicin O (2019). Emerging patient-specific treatment modalities in head and neck cancer—asystematic review. Expert Opin Investig Drugs.

[4] Amin MB (2017). The eighth edition AJCC cancer staging manual: continuing to build a bridge fro a population-based to a more “personalized” approach to cancer staging. CA Cancer J Clin.

[5] Angjelova A (2023). The potential of nano-based photodynamic treatment as a therapy against oral leukoplakia: a narrative review. J Clin Med.

[6] Arboleda L (2023). Squamous cell carcinoma of the oral cavity, oropharynx, and larynx: a scoping review of treatment guidelines worldwide. Cancers.

[7] Hajmohammadi E (2021). Sonodynamic therapy and common head and neck cancers: in vitro and in vivo studies. Eur Rev Med Pharmacol Sci.

[8] Park JO (2021). Survival benefits from surgery for stage IVa head and neck squamous cell carcinoma: a multi-institutional analysis of 1,033 cases. Clin Exp Otorhinolaryngol.

[9] Sacco AG, Cohen EE (2015). Current treatment options for recurrent or metastatic head and neck squamous cell carcinoma. J Clin Oncol.

[10] Mei Z (2020). Immune checkpoint pathways in immunotherapy for head and neck squamous cell carcinoma. Int J Oral Sci.

[11] Burcher KM (2021). Relationship between tumor mutational burden, PD-L1, patient characteristics, and response to immune checkpoint inhibitors in head and neck squamous cell carcinoma. Cancers.

[12] Shu T, Wang X (2023). Cuproptosis combines immune landscape providing prognostic biomarker in head and neck squamous carcinoma. Heliyon.

[13] Zhou L (2021). Epigenetic modulation of immunotherapy and implications in head and neck cancer. Cancer Metastasis Rev.

[14] Dos SE (2021). Epigenetic modulation of the tumor microenvironment in head and neck cancer: challenges and opportunities. Crit Rev Oncol Hematol.

[15] Van Speybroeck L (2002). From epigenesis to epigenetics: the case of C. H. Waddington. Ann N Y Acad Sci.

[16] Yu M (2020). Epigenetic aging: more than just a clock when it comes to cancer. Cancer Res.

[17] Chen Q (2022). Histone acetyltransferases CBP/p300 in tumorigenesis and CBP/p300 inhibitors as promising novel anticancer agents. Theranostics.

[18] Bradshaw PC (2021). Acetyl-CoA metabolism and histone acetylation in the regulation of aging and lifespan. Antioxidants.

[19] Yang L (2022). Histone deacetylase 3 facilitates TNFalpha-mediated NF-kappaB activation through suppressing CTSB induced RIP1 degradation and is required for host defense against bacterial infection. Cell Biosci.

[20] Millan-Zambrano G (2022). Histone post-translational modifications—cause and consequence of genome function. Nat Rev Genet.

[21] Verza FA (2020). Roles of histone deacetylases and inhibitors in anticancer therapy. Cancers.

[22] Banerjee DR (2019). Acetylation of the histone H3 tail domain regulates base excision repair on higher-order chromatin structures. Sci Rep.

[23] Dahlin JL (2015). Histone-modifying enzymes, histone modifications and histone chaperones in nucleosome assembly: lessons learned from Rtt109 histone acetyltransferases. Crit Rev Biochem Mol Biol.

[24] Popova LV (2021). Epigenetic regulation of nuclear lamina-associated heterochromatin by HAT1 and the acetylation of newly synthesized histones. Nucleic Acids Res.

[25] Singh M (2020). Histone acetyltransferase MOF orchestrates outcomes at the crossroad of oncogenesis, DNA damage response, proliferation, and stem cell development. Mol Cell Biol.

[26] Ramaiah MJ, Tangutur AD, Manyam RR (2021). Epigenetic modulation and understanding of HDAC inhibitors in cancer therapy. Life Sci.

[27] Yu X (2020). Natural HDAC-1/8 inhibitor baicalein exerts therapeutic effect in CBF-AML. Clin Transl Med.

[28] Yoshii H (2022). The expression of SIRT6 is associated with treatment outcome in elder patients with oral cancer. Anticancer Res.

[29] Kiesslich T, Neureiter D (2022). Can we efficiently target HDAC in cancer?. Cancers.

[30] Ho TCS, Chan AHY, Ganesan A (2020). Thirty years of HDAC inhibitors: 2020 insight and hindsight. J Med Chem.

[31] Wang TY (2021). Maintenance of HDACs and H3K9me3 prevents arterial flow-induced venous endothelial damage. Front Cell Dev Biol.

[32] Hu A (2015). Curcumin as therapeutics for the treatment of head and neck squamous cell carcinoma by activating SIRT1. Sci Rep.

[33] Milazzo G (2020). Histone deacetylases (HDACs): evolution, specificity, role in transcriptional complexes, and pharmacological actionability. Genes.

[34] Yang XJ, Seto E (2008). The Rpd3/Hda1 family of lysine deacetylases: from bacteria and yeast to mice and men. Nat Rev Mol Cell Biol.

[35] Watson PJ (2016). Insights into the activation mechanism of class I HDAC complexes by inositol phosphates. Nat Commun.

[36] Zhao R (2016). A correlation analysis between HDAC1 over-expression and clinical features of laryngeal squamous cell carcinoma. Acta Otolaryngol.

[37] Lv Y (2020). Histone deacetylase 1 regulates the malignancy of oral cancer cells via miR-154-5p/PCNA axis. Biol Chem.

[38] Park J (2019). CCL28-induced RARbeta expression inhibits oral squamous cell carcinoma bone invasion. J Clin Invest.

[39] Kondapuram SK, Coumar MS (2022). Pan-cancer gene expression analysis: identification of deregulated autophagy genes and drugs to target them. Gene.

[40] Lima DOJ (2022). Epithelial–mesenchymal transition and cancer stem cells: a route to acquired cisplatin resistance through epigenetics in HNSCC. Oral Dis.

[41] Chang HH (2009). Histone deacetylase 2 expression predicts poorer prognosis in oral cancer patients. Oral Oncol.

[42] Krishna A (2020). Upregulated histone deacetylase 2 gene correlates with the progression of oral squamous cell carcinoma. Cancer Biomark.

[43] Milan TM (2022). Epigenetic modifications control loss of adhesion and aggressiveness of cancer stem cells derived from head and neck squamous cell carcinoma with intrinsic resistance to cisplatin. Arch Oral Biol.

[44] Chang CC (2011). HDAC2 promotes cell migration/invasion abilities through HIF-1alpha stabilization in human oral squamous cell carcinoma. J Oral Pathol Med.

[45] Chen HL (2022). Trichodermin inhibits the growth of oral cancer through apoptosis-induced mitochondrial dysfunction and HDAC-2-mediated signaling. Biomed Pharmacother.

[46] Jou YJ (2015). Quantitative phosphoproteomic analysis reveals gamma-bisabolene inducing p53-mediated apoptosis of human oral squamous cell carcinoma via HDAC2 inhibition and ERK1/2 activation. Proteomics.

[47] Ramsey MR (2011). Physical association of HDAC1 and HDAC2 with p63 mediates transcriptional repression and tumor maintenance in squamous cell carcinoma. Cancer Res.

[48] Adhikari N, Jha T, Ghosh B (2021). Dissecting histone deacetylase 3 in multiple disease conditions: selective inhibition as a promising therapeutic strategy. J Med Chem.

[49] Chang H (2021). Lactate secreted by PKM2 upregulation promotes Galectin-9-mediated immunosuppression via inhibiting NF-kappaB pathway in HNSCC. Cell Death Dis.

[50] Amin SA, Adhikari N, Jha T (2017). Structure-activity relationships of hydroxamate-based histone deacetylase-8 inhibitors: reality behind anticancer drug discovery. Future Med Chem.

[51] Ahn MY, Yoon JH (2017). Histone deacetylase 8 as a novel therapeutic target in oral squamous cell carcinoma. Oncol Rep.

[52] Ahn MY (2018). HDAC inhibitor apicidin suppresses murine oral squamous cell carcinoma cell growth in vitro and in vivo via inhibiting HDAC8 expression. Oncol Lett.

[53] Liu L (2021). Targeting class IIa HDACs: insights from phenotypes and inhibitors. Curr Med Chem.

[54] Szigety KM (2020). HDAC3 ensures stepwise epidermal stratification via NCoR/SMRT-reliant mechanisms independent of its histone deacetylase activity. Genes Dev.

[55] Sun X (2023). HDAC4 mediated LHPP deacetylation enhances its destabilization and promotes the proliferation and metastasis of nasopharyngeal carcinoma. Cancer Lett.

[56] Schrenk C (2023). Synergistic interaction of the class IIa HDAC inhibitor CHDI0039 with bortezomib in head and neck cancer cells. Int J Mol Sci.

[57] Zeng LS (2016). Overexpressed HDAC4 is associated with poor survival and promotes tumor progression in esophageal carcinoma. Aging.

[58] Cheng C (2021). HDAC4 promotes nasopharyngeal carcinoma progression and serves as a therapeutic target. Cell Death Dis.

[59] Lu Z (2022). Promotion of microRNA-146a by histone deacetylase 4 silencing contributes to radiosensitization of esophageal carcinoma. J Transl Med.

[60] Lee BS (2018). HDAC4 degradation by combined TRAIL and valproic acid treatment induces apoptotic cell death of TRAIL-resistant head and neck cancer cells. Sci Rep.

[61] Heawchaiyaphum C (2023). The dual functions of andrographolide in the Epstein–Barr virus-positive head-and-neck cancer cells: the inhibition of lytic reactivation of the Epstein–Barr virus and the induction of cell death. Int J Mol Sci.

[62] Kai Y (2014). Reciprocal effects between microRNA-140-5p and ADAM10 suppress migration and invasion of human tongue cancer cells. Biochem Biophys Res Commun.

[63] Ahn MY, Yoon JH (2017). Histone deacetylase 7 silencing induces apoptosis and autophagy in salivary mucoepidermoid carcinoma cells. J Oral Pathol Med.

[64] Li QG (2020). HDAC7 promotes the oncogenicity of nasopharyngeal carcinoma cells by miR-4465-EphA2 signaling axis. Cell Death Dis.

[65] Wang Y (2023). Histone deacetylase 7: a signalling hub controlling development, inflammation, metabolism and disease. FEBS J.

[66] Ning Y (2020). HDAC9 deficiency promotes tumor progression by decreasing the CD8(+) dendritic cell infiltration of the tumor microenvironment. J Immunother Cancer.

[67] Rastogi B (2016). Overexpression of HDAC9 promotes oral squamous cell carcinoma growth, regulates cell cycle progression, and inhibits apoptosis. Mol Cell Biochem.

[68] Rastogi B (2017). Downregulation of miR-377 promotes oral squamous cell carcinoma growth and migration by targeting HDAC9. Cancer Invest.

[69] Sakuma T (2006). Aberrant expression of histone deacetylase 6 in oral squamous cell carcinoma. Int J Oncol.

[70] Kaur S, Rajoria P, Chopra M (2022). HDAC6: a unique HDAC family member as a cancer target. Cell Oncol.

[71] Wang XC (2015). miR-433 inhibits oral squamous cell carcinoma (OSCC) cell growth and metastasis by targeting HDAC6. Oral Oncol.

[72] Liu F (2017). MiR-206 inhibits Head and neck squamous cell carcinoma cell progression by targeting HDAC6 via PTEN/AKT/mTOR pathway. Biomed Pharmacother.

[73] Tseng CC (2022). HDAC6 is a prognostic biomarker that mediates IL-13 expression to regulate macrophage polarization through AP-1 in oral squamous cell carcinoma. Sci Rep.

[74] Chang I, Wang CY (2016). Inhibition of HDAC6 protein enhances bortezomib-induced apoptosis in head and neck squamous cell carcinoma (HNSCC) by reducing autophagy. J Biol Chem.

[75] Tavares MO (2022). Pharmacological inhibition of HDAC6 overcomes cisplatin chemoresistance by targeting cancer stem cells in oral squamous cell carcinoma. J Oral Pathol Med.

[76] Cheng F (2021). Histone deacetylase 10, a potential epigenetic target for therapy. Biosci Rep.

[77] Dai H (2018). Sirtuin activators and inhibitors: promises, achievements, and challenges. Pharmacol Ther.

[78] Islam S (2019). Sirtuin 1 and oral cancer. Oncol Lett.

[79] Navas LE, Carnero A (2021). NAD(+) metabolism, stemness, the immune response, and cancer. Signal Transduct Target Ther.

[80] Wu QJ (2022). The sirtuin family in health and disease. Signal Transduct Target Ther.

[81] Kang YY (2018). SIRT1 acts as a potential tumor suppressor in oral squamous cell carcinoma. J Chin Med Assoc.

[82] Ezhilarasan D (2022). The ambiguous role of sirtuins in head and neck squamous cell carcinoma. Oral Dis.

[83] Murofushi T (2017). CAY10591, a SIRT1 activator, suppresses cell growth, invasion, and migration in gingival epithelial carcinoma cells. J Oral Sci.

[84] Chen IC (2014). Role of SIRT1 in regulation of epithelial-to-mesenchymal transition in oral squamous cell carcinoma metastasis. Mol Cancer.

[85] Vaiciulis P (2022). Association of SIRT1 single gene nucleotide polymorphisms and serum SIRT1 levels with laryngeal squamous cell carcinoma patient survival rate. Cancer Biomark.

[86] Noguchi A (2013). SIRT1 expression is associated with good prognosis for head and neck squamous cell carcinoma patients. Oral Surg Oral Med Oral Pathol Oral Radiol.

[87] Xiong P (2011). Proteomic analyses of Sirt1-mediated cisplatin resistance in OSCC cell line. Protein J.

[88] Ye Z (2021). MiR-34a reverses radiation resistance on ECA-109 cells by inhibiting PI3K/AKT/mTOR signal pathway through downregulating the expression of SIRT1. Int J Radiat Biol.

[89] Ai J (2023). Exosomes loaded with circPARD3 promotes EBV-miR-BART4-induced stemness and cisplatin resistance in nasopharyngeal carcinoma side population cells through the miR-579-3p/SIRT1/SSRP1 axis. Cell Biol Toxicol.

[90] Chang CF (2020). Capsaicin acts through tNOX (ENOX2) to induce autophagic apoptosis in p53-mutated HSC-3 cells but autophagy in p53-functional SAS oral cancer cells. Am J Cancer Res.

[91] Chen G, Huang P, Hu C (2020). The role of SIRT2 in cancer: a novel therapeutic target. Int J Cancer.

[92] O'Callaghan C, Vassilopoulos A (2017). Sirtuins at the crossroads of stemness, aging, and cancer. Aging Cell.

[93] Hu A (2020). SIRT2 modulates VEGFD-associated lymphangiogenesis by deacetylating EPAS1 in human head and neck cancer. Mol Carcinog.

[94] Alhazzazi TY (2016). A novel sirtuin-3 inhibitor, LC-0296, inhibits cell survival and proliferation, and promotes apoptosis of head and neck cancer cells. Anticancer Res.

[95] Chen IC (2013). Role of SIRT3 in the regulation of redox balance during oral carcinogenesis. Mol Cancer.

[96] Yang M, Yang C, Pei Y (2014). Effects of downregulation of SIRT3 expression on proliferation and apoptosis in esophageal squamous cell carcinoma EC9706 cells and its molecular mechanisms. Biomed Mater Eng.

[97] Ansari A (2017). Function of the SIRT3 mitochondrial deacetylase in cellular physiology, cancer, and neurodegenerative disease. Aging Cell.

[98] Mahjabeen I, Kayani MA (2016). Loss of mitochondrial tumor suppressor genes expression is associated with unfavorable clinical outcome in head and neck squamous cell carcinoma: data from retrospective study. PLoS ONE.

[99] Kao YY (2019). MicroRNA miR-31 targets SIRT3 to disrupt mitochondrial activity and increase oxidative stress in oral carcinoma. Cancer Lett.

[100] Tao L, Park JY, Lambert JD (2015). Differential prooxidative effects of the green tea polyphenol, (−)-epigallocatechin-3-gallate, in normal and oral cancer cells are related to differences in sirtuin 3 signaling. Mol Nutr Food Res.

[101] Alhazzazi TY (2011). Sirtuin-3 (SIRT3), a novel potential therapeutic target for oral cancer. Cancer.

[102] Huang G, Zhu G (2018). Sirtuin-4 (SIRT4), a therapeutic target with oncogenic and tumor-suppressive activity in cancer. Onco Targets Ther.

[103] Jeong SM (2013). SIRT4 has tumor-suppressive activity and regulates the cellular metabolic response to DNA damage by inhibiting mitochondrial glutamine metabolism. Cancer Cell.

[104] Wan W (2020). SIRT4 expression in laryngeal squamous cell carcinoma. Pharmazie.

[105] Lagunas-Rangel FA (2023). Role of SIRT5 in cancer. Friend or Foe?. Biochimie.

[106] Deng SZ (2023). Integrative analysis of lysine acetylation-related genes and identification of a novel prognostic model for oral squamous cell carcinoma. Front Mol Biosci.

[107] Lu CT (2014). The potential of SIRT6 and SIRT7 as circulating markers for head and neck squamous cell carcinoma. Anticancer Res.

[108] Sripodok P (2023). Immunoexpression of SIRT1, 6, and 7 in oral leukoplakia and oral squamous cell carcinoma. Odontology.

[109] Lefort K (2013). A miR-34a-SIRT6 axis in the squamous cell differentiation network. EMBO J.

[110] Park JJ (2021). MDM2-dependent Sirt1 degradation is a prerequisite for Sirt6-mediated cell death in head and neck cancers. Exp Mol Med.

[111] Ouyang L (2018). SIRT6 overexpression induces apoptosis of nasopharyngeal carcinoma by inhibiting NF-kappaB signaling. Onco Targets Ther.

[112] Li W, Zhu D, Qin S (2018). SIRT7 suppresses the epithelial-to-mesenchymal transition in oral squamous cell carcinoma metastasis by promoting SMAD4 deacetylation. J Exp Clin Cancer Res.

[113] Jia B (2021). MiR-770 promotes oral squamous cell carcinoma migration and invasion by regulating the Sirt7/Smad4 pathway. IUBMB Life.

[114] Malik S (2015). SIRT7 inactivation reverses metastatic phenotypes in epithelial and mesenchymal tumors. Sci Rep.

[115] Liu SS (2020). HDAC11: a rising star in epigenetics. Biomed Pharmacother.

[116] Hassig CA (1997). Histone deacetylase activity is required for full transcriptional repression by mSin3A. Cell.

[117] Haberland M (2009). Genetic dissection of histone deacetylase requirement in tumor cells. Proc Natl Acad Sci USA.

[118] Segre CV, Chiocca S (2011). Regulating the regulators: the post-translational code of class I HDAC1 and HDAC2. J Biomed Biotechnol.

[119] Johnson DE (2020). Head and neck squamous cell carcinoma. Nat Rev Dis Primers.

[120] Mann BS (2007). FDA approval summary: vorinostat for treatment of advanced primary cutaneous T-cell lymphoma. Oncologist.

[121] Whittaker SJ (2010). Final results from a multicenter, international, pivotal study of romidepsin in refractory cutaneous T-cell lymphoma. J Clin Oncol.

[122] Coiffier B (2012). Results from a pivotal, open-label, phase II study of romidepsin in relapsed or refractory peripheral T-cell lymphoma after prior systemic therapy. J Clin Oncol.

[123] McDermott J, Jimeno A (2014). Belinostat for the treatment of peripheral T-cell lymphomas. Drugs Today.

[124] Lee P (2015). Mechanisms and clinical significance of histone deacetylase inhibitors: epigenetic glioblastoma therapy. Anticancer Res.

[125] Finnin MS (1999). Structures of a histone deacetylase homologue bound to the TSA and SAHA inhibitors. Nature.

[126] Bhansali P (2014). Synthesis and biological evaluation of largazole analogues with modified surface recognition cap groups. Eur J Med Chem.

[127] Tessier P (2009). Diphenylmethylene hydroxamic acids as selective class IIa histone deacetylase inhibitors. Bioorg Med Chem Lett.

[128] Friend C (1971). Hemoglobin synthesis in murine virus-induced leukemic cells in vitro: stimulation of erythroid differentiation by dimethyl sulfoxide. Proc Natl Acad Sci USA.

[129] Marks PA, Breslow R (2007). Dimethyl sulfoxide to vorinostat: development of this histone deacetylase inhibitor as an anticancer drug. Nat Biotechnol.

[130] Zhang L (2018). Zinc binding groups for histone deacetylase inhibitors. J Enzyme Inhib Med Chem.

[131] Jang B (2016). Growth-suppressive effect of suberoylanilide hydroxamic acid (SAHA) on human oral cancer cells. Cell Oncol.

[132] Kakiuchi A (2021). HDAC inhibitors suppress the proliferation, migration and invasiveness of human head and neck squamous cell carcinoma cells via p63mediated tight junction molecules and p21mediated growth arrest. Oncol Rep.

[133] Cheng YW (2018). The histone deacetylase inhibitor panobinostat exerts anticancer effects on esophageal squamous cell carcinoma cells by inducing cell cycle arrest. Cell Biochem Funct.

[134] Ushio R (2022). Enhanced cytotoxic effects in human oral squamous cell carcinoma cells treated with combined methyltransferase inhibitors and histone deacetylase inhibitors. Biomedicines.

[135] Liu S (2017). Nuclear survivin promoted by acetylation is associated with the aggressive phenotype of oral squamous cell carcinoma. Cell Cycle.

[136] Eriksson I (2013). The histone deacetylase inhibitor trichostatin A reduces lysosomal pH and enhances cisplatin-induced apoptosis. Exp Cell Res.

[137] Jia L (2017). Trichostatin A increases radiosensitization of tongue squamous cell carcinoma via miR-375. Oncol Rep.

[138] Atadja P (2009). Development of the pan-DAC inhibitor panobinostat (LBH589): successes and challenges. Cancer Lett.

[139] Shao W (2010). Activity of deacetylase inhibitor panobinostat (LBH589) in cutaneous T-cell lymphoma models: defining molecular mechanisms of resistance. Int J Cancer.

[140] Giles F (2006). A phase I study of intravenous LBH589, a novel cinnamic hydroxamic acid analogue histone deacetylase inhibitor, in patients with refractory hematologic malignancies. Clin Cancer Res.

[141] Jeon YJ (2013). The HDAC inhibitor, panobinostat, induces apoptosis by suppressing the expresssion of specificity protein 1 in oral squamous cell carcinoma. Int J Mol Med.

[142] Venugopal B (2013). A phase I study of quisinostat (JNJ-26481585), an oral hydroxamate histone deacetylase inhibitor with evidence of target modulation and antitumor activity, in patients with advanced solid tumors. Clin Cancer Res.

[143] Wang X (2021). Death by histone deacetylase inhibitor quisinostat in tongue squamous cell carcinoma via apoptosis, pyroptosis, and ferroptosis. Toxicol Appl Pharmacol.

[144] Kuribayashi T (2010). Scriptaid, a novel histone deacetylase inhibitor, enhances the response of human tumor cells to radiation. Int J Mol Med.

[145] Choi E (2012). Property-based optimization of hydroxamate-based γ-lactam HDAC inhibitors to improve their metabolic stability and pharmacokinetic profiles. J Med Chem.

[146] Shin JA (2014). The in vitro apoptotic effects of A248 and A1659, newly synthetic histone deacetylase inhibitors in oral cancer cells. Oral Dis.

[147] Cao J (2018). Ricolinostat (ACY-1215) suppresses proliferation and promotes apoptosis in esophageal squamous cell carcinoma via miR-30d/PI3K/AKT/mTOR and ERK pathways. Cell Death Dis.

[148] Pulya S (2021). HDAC6 as privileged target in drug discovery: a perspective. Pharmacol Res.

[149] Miyake K (2022). Ricolinostat enhances adavosertib-induced mitotic catastrophe in TP53-mutated head and neck squamous cell carcinoma cells. Int J Oncol.

[150] Hattori K (2021). Induction of synergistic non-apoptotic cell death by simultaneously targeting proteasomes with bortezomib and histone deacetylase 6 with ricolinostat in head and neck tumor cells. Oncol Lett.

[151] Sun S (2021). Design, synthesis and antitumor activity evaluation of novel HDAC inhibitors with tetrahydrobenzothiazole as the skeleton. Bioorg Chem.

[152] Mahal K (2015). Biological evaluation of 4,5-diarylimidazoles with hydroxamic acid appendages as novel dual mode anticancer agents. Cancer Chemother Pharmacol.

[153] Miller TA (2005). Patent status of histone deacetylase inhibitors. Expert Opin Ther Pat.

[154] Haigentz MJ (2012). Phase II trial of the histone deacetylase inhibitor romidepsin in patients with recurrent/metastatic head and neck cancer. Oral Oncol.

[155] Sasaki Y (2008). Histone deacetylase inhibitor FK228 enhances adenovirus-mediated p53 family gene therapy in cancer models. Mol Cancer Ther.

[156] Bauden M, Tassidis H, Ansari D (2015). In vitro cytotoxicity evaluation of HDAC inhibitor apicidin in pancreatic carcinoma cells subsequent time and dose dependent treatment. Toxicol Lett.

[157] Taori K, Paul VJ, Luesch H (2008). Structure and activity of largazole, a potent antiproliferative agent from the Floridian marine cyanobacterium *Symploca* sp.. J Am Chem Soc.

[158] Ying Y (2008). Total synthesis and molecular target of largazole, a histone deacetylase inhibitor. J Am Chem Soc.

[159] Poli G (2017). Largazole analogues as histone deacetylase inhibitors and anticancer agents: an overview of structure–activity relationships. ChemMedChem.

[160] Zhang B (2020). Unexpected enhancement of HDACs inhibition by MeS substitution at C-2 position of fluoro largazole. Mar Drugs.

[161] Krieger V (2017). Design, multicomponent synthesis, and anticancer activity of a focused histone deacetylase (HDAC) inhibitor library with peptoid-based cap groups. J Med Chem.

[162] Jung M (2005). Novel HDAC inhibitors with radiosensitizing properties. Radiat Res.

[163] Amin SA (2019). Histone deacetylase 3 inhibitors in learning and memory processes with special emphasis on benzamides. Eur J Med Chem.

[164] El-Beltagi HM (1993). Acetyldinaline: a new oral cytostatic drug with impressive differential activity against leukemic cells and normal stem cells–preclinical studies in a relevant rat model for human acute myelocytic leukemia. Cancer Res.

[165] Seelig MH, Berger MR (1996). Efficacy of dinaline and its methyl and acetyl derivatives against colorectal cancer in vivo and in vitro. Eur J Cancer.

[166] Connolly RM (2017). Combination epigenetic therapy in advanced breast cancer with 5-azacitidine and entinostat: a phase II National Cancer Institute/stand up to cancer study. Clin Cancer Res.

[167] Jespersen H (2019). Concomitant use of pembrolizumab and entinostat in adult patients with metastatic uveal melanoma (PEMDAC study): protocol for a multicenter phase II open label study. BMC Cancer.

[168] Marques A (2020). Entinostat is a novel therapeutic agent to treat oral squamous cell carcinoma. J Oral Pathol Med.

[169] Liu X (2023). Chidamide, a novel histone deacetylase inhibitor, inhibits laryngeal cancer progression in vitro and in vivo. Int J Biochem Cell Biol.

[170] De Schutter H (2009). A systematic assessment of radiation dose enhancement by 5-Aza-2′-deoxycytidine and histone deacetylase inhibitors in head-and-neck squamous cell carcinoma. Int J Radiat Oncol Biol Phys.

[171] He Y (2019). Metformin and 4SC-202 synergistically promote intrinsic cell apoptosis by accelerating DeltaNp63 ubiquitination and degradation in oral squamous cell carcinoma. Cancer Med.

[172] He Y (2020). Metformin combined with 4SC-202 inhibited the migration and invasion of OSCC via STAT3/TWIST1. Onco Targets Ther.

[173] Yang X (2022). 4sc-202 and Ink-128 cooperate to reverse the epithelial to mesenchymal transition in OSCC. Oral Dis.

[174] Liang X (2019). Combined class I histone deacetylase and mTORC1/C2 inhibition suppresses the initiation and recurrence of oral squamous cell carcinomas by repressing SOX2. Cancer Lett.

[175] Damm JK (2015). Pharmacologically relevant doses of valproate upregulate CD20 expression in three diffuse large B-cell lymphoma patients in vivo. Exp Hematol Oncol.

[176] Gore SD, Samid D, Weng LJ (1997). Impact of the putative differentiating agents sodium phenylbutyrate and sodium phenylacetate on proliferation, differentiation, and apoptosis of primary neoplastic myeloid cells. Clin Cancer Res.

[177] Wawruszak A (2021). Valproic acid and breast cancer: state of the art in 2021. Cancers.

[178] López-Verdín S (2018). Molecular markers of anticancer drug resistance in head and neck squamous cell carcinoma: a literature review. Cancers.

[179] Iannelli F (2020). Valproic acid synergizes with cisplatin and cetuximab in vitro and in vivo in head and neck cancer by targeting the mechanisms of resistance. Front Cell Dev Biol.

[180] Yue HQ (2022). Sodium butyrate inhibits oral squamous cell carcinoma proliferation and invasion by regulating the HDAC1/HSPB7 axis. Neoplasma.

[181] Mrkvicova A (2019). The effect of sodium butyrate and cisplatin on expression of EMT markers. PLoS ONE.

[182] Zang W (2022). Butyrate promotes oral squamous cell carcinoma cells migration, invasion and epithelial–mesenchymal transition. PeerJ.

[183] Ohshima M (2017). Metabolomic analysis of the saliva of Japanese patients with oral squamous cell carcinoma. Oncol Rep.

[184] Tsai CK (2020). Nuclear magnetic resonance metabolomics biomarkers for identifying high risk patients with extranodal extension in oral squamous cell carcinoma. J Clin Med.

[185] Bai LY (2011). Antitumor activity of a novel histone deacetylase inhibitor (S)-HDAC42 in oral squamous cell carcinoma. Oral Oncol.

[186] Bai LY (2011). Antitumor effects of (S)-HDAC42, a phenylbutyrate-derived histone deacetylase inhibitor, in multiple myeloma cells. Cancer Chemother Pharmacol.

[187] Hehlgans S (2013). The novel HDAC inhibitor NDACI054 sensitizes human cancer cells to radiotherapy. Radiother Oncol.

[188] Marek L (2013). Histone deacetylase (HDAC) inhibitors with a novel connecting unit linker region reveal a selectivity profile for HDAC4 and HDAC5 with improved activity against chemoresistant cancer cells. J Med Chem.

[189] Choi SY (2018). Inhibition of class IIa histone deacetylase activity by gallic acid, sulforaphane, TMP269, and panobinostat. Biomed Pharmacother.

[190] Asfaha Y (2020). Novel alkoxyamide-based histone deacetylase inhibitors reverse cisplatin resistance in chemoresistant cancer cells. Bioorg Med Chem.

[191] Galloway TJ (2015). A phase I study of CUDC-101, a multitarget inhibitor of HDACs, EGFR, and HER2, in combination with chemoradiation in patients with head and neck squamous cell carcinoma. Clin Cancer Res.

[192] Lai CJ (2010). CUDC-101, a multitargeted inhibitor of histone deacetylase, epidermal growth factor receptor, and human epidermal growth factor receptor 2, exerts potent anticancer activity. Cancer Res.

[193] Babar Q (2022). Novel epigenetic therapeutic strategies and targets in cancer. Biochim Biophys Acta Mol Basis Dis.

[194] Ling R (2023). HDAC—an important target for improving tumor radiotherapy resistance. Front Oncol.

[195] Teknos TN (2019). A phase 1 trial of Vorinostat in combination with concurrent chemoradiation therapy in the treatment of advanced staged head and neck squamous cell carcinoma. Invest New Drugs.

[196] Mak MP (2020). Valproic acid combined with cisplatin-based chemoradiation in locally advanced head and neck squamous cell carcinoma patients and associated biomarkers. Ecancermedicalscience.

[197] Cameron EE (1999). Synergy of demethylation and histone deacetylase inhibition in the re-expression of genes silenced in cancer. Nat Genet.

[198] Silva LC (2023). Repurposing NFkappaB and HDAC inhibitors to individually target cancer stem cells and non-cancer stem cells from mucoepidermoid carcinomas. Am J Cancer Res.

[199] Iwasa YI (2023). A spatial transcriptome reveals changes in tumor and tumor microenvironment in oral cancer with acquired resistance to immunotherapy. Biomolecules.

[200] Gray JE (2014). A phase I, pharmacokinetic, and pharmacodynamic study of panobinostat, an HDAC inhibitor, combined with erlotinib in patients with advanced aerodigestive tract tumors. Clin Cancer Res.

[201] Rodriguez CP (2020). A phase II trial of pembrolizumab and vorinostat in recurrent metastatic head and neck squamous cell carcinomas and salivary gland cancer. Clin Cancer Res.

[202] Mahvi DA (2018). Local cancer recurrence: the realities, challenges, and opportunities for new therapies. CA Cancer J Clin.

[203] Peinado H (2017). Pre-metastatic niches: organ-specific homes for metastases. Nat Rev Cancer.

[204] Lu Z (2020). Epigenetic therapy inhibits metastases by disrupting premetastatic niches. Nature.

[205] Wykrzykowska JJ, Bianchi C, Sellke FW (2009). Impact of aging on the angiogenic potential of the myocardium: implications for angiogenic therapies with emphasis on sirtuin agonists. Recent Pat Cardiovasc Drug Discov.

[206] Howitz KT (2003). Small molecule activators of sirtuins extend Saccharomyces cerevisiae lifespan. Nature.

[207] Milne JC (2007). Small molecule activators of SIRT1 as therapeutics for the treatment of type 2 diabetes. Nature.

