# Supplementary figures and images for: Targeting histone deacetylases in head and neck squamous cell carcinoma: molecular mechanisms and therapeutic targets
Source: J Transl Med. 2024 May 3;22:418. doi: 10.1186/s12967-024-05169-9 (PMC11067317; doi:10.1186/s12967-024-05169-9)

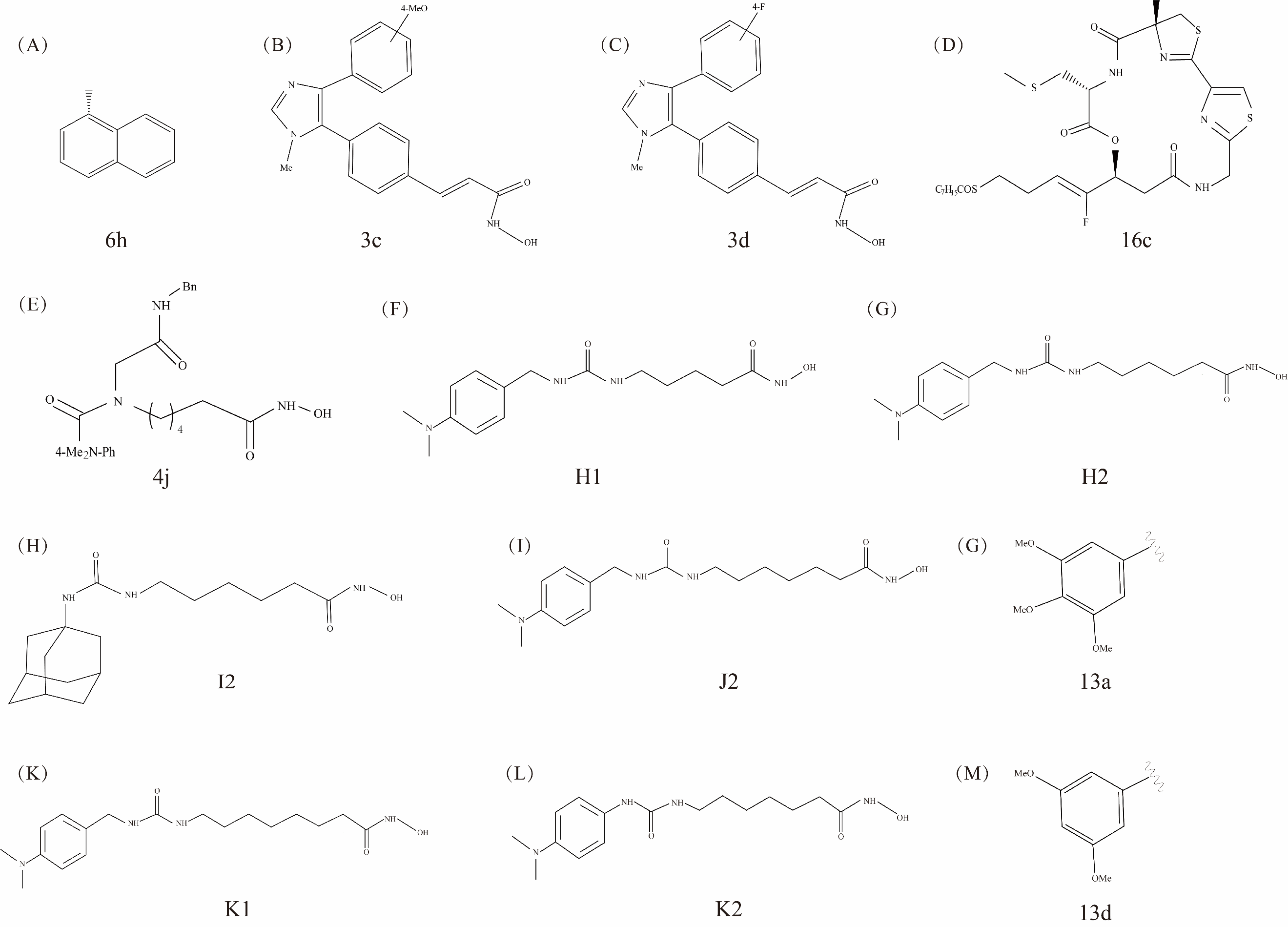


Figure S1. The molecular structures of novel HDAC inhibitors.

Supplement: Supplementary file 1 — Additional file 1: Figure S1. The molecular structures of novel HDAC inhibitors. [file 12967_2024_5169_MOESM1_ESM.docx]
